# Plant organellar RNA maturation

**DOI:** 10.1093/plcell/koad049

**Published:** 2023-02-21

**Authors:** Ian Small, Joanna Melonek, Alexandra-Viola Bohne, Jörg Nickelsen, Christian Schmitz-Linneweber

**Affiliations:** Australian Research Council Centre of Excellence in Plant Energy Biology, School of Molecular Sciences, The University of Western Australia, Crawley 6009, Australia; Australian Research Council Centre of Excellence in Plant Energy Biology, School of Molecular Sciences, The University of Western Australia, Crawley 6009, Australia; Department of Molecular Plant Sciences, LMU Munich, 82152 Martinsried, Germany; Department of Molecular Plant Sciences, LMU Munich, 82152 Martinsried, Germany; Molecular Genetics, Humboldt University Berlin, Philippstr. 13, 10115 Berlin, Germany

## Abstract

Plant organellar RNA metabolism is run by a multitude of nucleus-encoded RNA-binding proteins (RBPs) that control RNA stability, processing, and degradation. In chloroplasts and mitochondria, these post-transcriptional processes are vital for the production of a small number of essential components of the photosynthetic and respiratory machinery—and consequently for organellar biogenesis and plant survival. Many organellar RBPs have been functionally assigned to individual steps in RNA maturation, often specific to selected transcripts. While the catalog of factors identified is ever-growing, our knowledge of how they achieve their functions mechanistically is far from complete. This review summarizes the current knowledge of plant organellar RNA metabolism taking an RBP-centric approach and focusing on mechanistic aspects of RBP functions and the kinetics of the processes they are involved in.

## Introduction

### Essential complexity in plant organellar RNA maturation

Chloroplasts and mitochondria have their own, relatively small, genomes. However, both genomes carry particularly important genes associated with photosynthesis and respiration. Indeed, these genes are essential for the survival of the entire organism, be it plant or algae. As descendants of bacterial ancestors, organellar gene organization is characterized by prokaryotic features. Genes are often arranged in operons, and many expression signals are of prokaryotic origin, such as the Shine–Dalgarno sequence for translation initiation. However, several billion years residing in eukaryotic cells has transformed the expression of organellar genes in chloroplasts and mitochondria. New and surprising events occur at all levels of gene expression. This is especially true for the steps between RNA synthesis and translation. Unlike cyanobacteria, plants and algae engage in complex maturation of organellar RNA. RNA precursors are processed into many segments, which require a set of RNases on one hand (Stoppel and Meurer 2011) and an array of RNA-binding proteins (RBPs) as protective factors on the other. The interplay of the RNases and RBPs leads to extremely complex transcript patterns in plant organelles. Also, RBPs render plant organellar transcripts stable over long periods of time. The half-lives of organellar RNAs are in the range of hours (Germain et al. 2013), whereas typical mRNAs in prokaryotes last only minutes (Selinger et al. 2003; Kristoffersen et al. 2012). Other peculiarities include introns, which are much more abundant in plant organelles compared to bacteria per kilobase of genetic information (Miura et al. 2022), and RNA editing, which is unknown in bacteria but can take epic dimensions in organelles, with nearly 500 edited sites in the organelles of Arabidopsis (*Arabidopsis thaliana*; Small et al. 2020) and 3,400 sites in the chloroplasts of the lycophyte *Selaginella uncinata* (Oldenkott et al. 2014). However, editing evolved in land plants, and to date, no organellar editing has been observed in green algae (Ichinose and Sugita 2017). In recent decades, a variety of RBPs have been characterized that are involved in individual RNA maturation steps. More RBPs are present per gene in plant organelles than in any other known genetic compartment. To what extent these RBPs have a role in organellar gene regulatory processes is at present unclear.

### A multitude of nucleus-encoded proteins is required for a variety of organellar RNA maturation events

**Figure 1.**
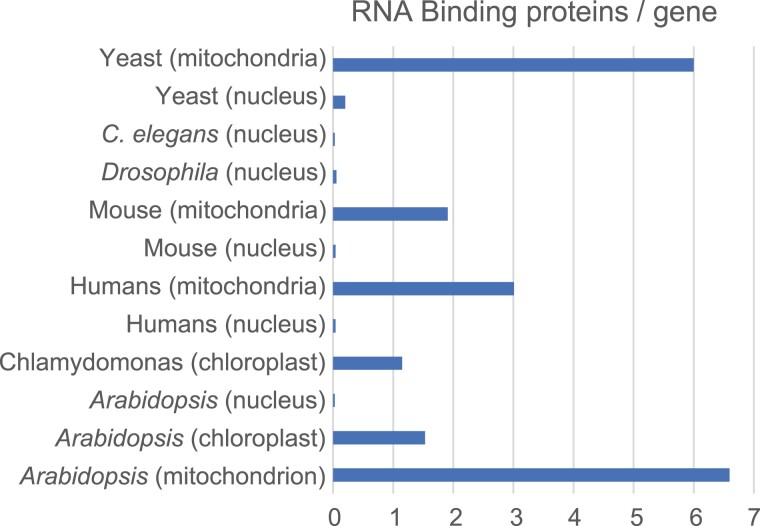
Abundance of RBPs in different organisms. RBP numbers are set in relation to gene numbers for humans and several model species as well as chloroplasts of *Arabidopsis* as a representative of embryophytes. For gene and RBP numbers as well as references, see Supplemental Table S1.

RNA maturation requires both catalytically active factors, such as RNases, and factors that confer specificity to RNA maturation events, such as RBPs. RBPs are responsible for virtually every aspect of the life cycle of an RNA, including maturation, stabilization, localization, translation, and degradation (see also the review by Mateos and Staiger 2023, in this issue). Based on genome annotations, localization predictions using the SUBA4 database for Arabidopsis proteins, and published literature, approximately 550 nucleus-encoded RBPs are conservatively estimated to be present in plant organelles (Table 1). Among them, the majority of RBPs belong to the pentatricopeptide repeat (PPR) protein family, with 106 and 328 RBPs predicted to be found in chloroplasts and mitochondria, respectively (Lurin et al. 2004; Hooper et al. 2014). In Chlamydomonas (*Chlamydomonas reinhardtii*) chloroplasts, at least 139 nuclear-encoded RBPs from the PPR (Tourasse et al. 2013), octotricopeptide repeat (OPR) (Cerutti, Bohne, Rochaix, Vallon, unpublished data), and TRP-HAT (Bohne et al. 2016) family are found. At first glance, 550 RBPs is not an overly impressive number in the age of genomics. After all, hundreds of RBPs are found in yeast, mice, and other model organisms (Keene 2001; Lee and Schedl 2006; Kerner et al. 2011). In humans, more than 1,500 RBPs have been identified using mRNA capture techniques (Baltz et al. 2012; Castello et al. 2012). Nevertheless, the number of RBPs found in chloroplasts is astounding in relation to the number of genes they serve. For example, Arabidopsis chloroplasts have 120 genes, with more than one RBP present per gene, whereas human nuclei have 22,000 genes with an RBP/gene ratio of 0.07 (Fig. 1 and Supplemental Table S1). It can be argued that a minimal set of RBPs is required to drive gene expression (e.g. to make a ribosome) even if only a single protein is produced. Thus, the RBP/gene ratio is high for mitochondria in mammals and yeast considering their limited coding potential (Fig. 1). A difference between the numbers shown here for plant and human/yeast organelles is that the human and yeast data are based on protein–RNA interactome studies, which led to the discovery of a multitude of noncanonical RBPs, while the plant organellar RBPs are based on canonical RBP annotations with standard RNA-binding domains. It can be expected that future similar discoveries of nonstandard RBPs in plant organelles will further increase the number of chloroplast and mitochondrial RBPs in plants and alga.

**Table 1.** RBP families in Arabidopsis chloroplasts and mitochondria

Protein family	Number of proteins targeted to chloroplasts^a^	Number of proteins targeted to mitochondria^a^	References
Pentatricopeptide repeat (PPR) proteins	106	328	Supplemental Table S4 in Lurin et al. (2004)
Chloroplast RNA splicing and ribosome maturation (CRM)—proteins	10	5	Barkan et al. (2007) and Zhang et al. (2021)
Whirly (WHY)-proteins	2	1	Prikryl et al. (2008)
Plant organellar RNA recognition (PORR)-proteins	3	11	Kroeger et al. (2009) andFrancs-Small et al. (2012)
DUF794/APO1	2	2	Watkins et al. (2011)
cpRNPs—chloroplast ribonucleoproteins (10)	10	0	Ruwe et al. (2011)
RNA recognition motif proteins (non-cpRNPs)	13	10	Ruwe et al. (2011)
Dead box helicases	11	8	Bobik et al. (2017) and Nawaz and Kang (2017)
RNAse III-like: RNC1	1	0	Watkins et al. (2007)
Peptidyl-tRNA hydrolase: CRS2	1	0	Jenkins and Barkan (2001)
Ribosome-release factors:	3	0	Stoppel et al. (2011)
Tetratricopeptide repeat protein binding RNA (R-TPR)/HCF107	1	0	Sane et al. (2005) and Hammani et al. (2012)
Mitochondrial transcription termination factor (mTERF)—proteins	12	10	Kleine (2012) and Robles and Quesada (2021)
K-homology (KH) domain proteins	3	1	SUBA
All RBP	185	276	

Number of proteins is based on SUBA-predictions (Hooper et al. 2014), which incorporate mass-spec. data as well as microscopic localizations. In addition, numbers from references indicated in the last column are considered as well.

### Ribonucleases: agonists of RNA decay and processing

The joint action of RBPs and Rnases determines organellar RNA turnover and transcript ends. The RNases that cleave organellar transcripts include endonucleases RNase E, CSP41a, CSP41b, and RNase J in chloroplasts (Stoppel and Meurer 2011; Germain et al. 2013) and PRORP (RNaseP), MNU1 and MNU2 in mitochondria (Gutmann et al. 2012; Stoll and Binder 2016; Bouchoucha et al. 2019). RNase E, and possibly RNase J, were shown to prefer A/U-rich sequences in vitro (Schein et al. 2008; Halpert et al. 2019), matching the preference for AU-rich organellar genomes, particularly AU-rich intergenic spacers, which provide ample opportunities for RNA cleavage. However, whether RNA cleavage occurs at hotspots or shows specificity in vivo remains unknown. Interaction studies using endonucleases with RNA-plus-degradome sequencing are needed to elucidate the initiation of RNA degradation.

**Figure 2.**
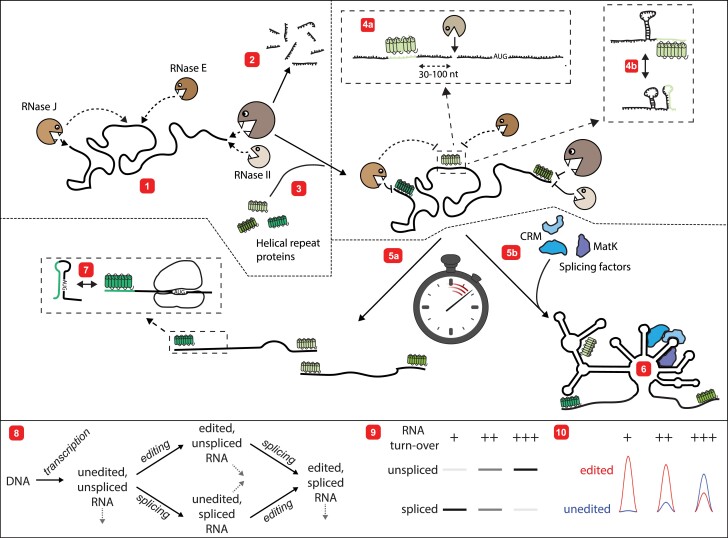
Helical repeat proteins extend half-lives of organellar mRNAs. 1, Organellar RNAs are under attack by a variety of RNases (e.g. RNAse J, Rnase E, RNase II, and PNPase) during and after transcription. 2, If not protected, RNAs decay rapidly. 3, Helical repeat proteins protect RNAs against exo- and endonucleases. 4a, In mitochondria, PPR proteins bind to 5′ UTR regions and guide endonucleolytic cleavage, for instance via RNase P. 4b, Binding of helical repeat proteins to RNAs can also impact the local RNA structure since they keep their binding site in a single-stranded conformation. This can, for example, aid in intron folding and thus splicing. 5, The protective action of helical repeat proteins increases target RNA half-lives and thus opens an extended window of opportunity for translation and other RNA processing events, for example, splicing. 5a, Stochastic endonucleolytic cuts initiate exonucleolytic degradation up to helical repeat proteins, which leads to the complex transcript patterns known from plant organelles. Adjacent RNAs overlap at helical repeat protein BS. 5b and 6, The stabilization of RNAs allows slow processes like group II intron folding to happen. Splicing factors like CRM or MatK have time to aid in folding as RNA chaperones to eventually complete the catalytically active intron structure. 7, Helical repeat proteins remodel RNA structures in 5′-UTRs to allow ribosome entry at start codons. 8, flowchart of RNA editing and RNA splicing in plant organelles. Four RNA species can be distinguished for any RNA that is both spliced and edited. The gray arrows indicate that all RNA isoforms are subject to RNA degradation. This flowchart formed the basis for a mathematical model (using Catalyst.jl, Loman et al. 2022) to explore the effects of different RNA turnover rates on the splicing and editing status. 9 and 10, Ratios of spliced to unspliced and edited to unedited RNAs are shown at different degradation rates as electronic RNA gel blot results and modeled Sanger sequencing results. Each “+” indicates an order of magnitude increase in RNA degradation rate.

Once a free transcript end has been generated, polyadenylation by organelle poly (A) polymerases triggers exonucleolytic degradation (Zimmer et al. 2009; Hirayama 2021), which is performed by poly (A)-specific 3′–5′ exonucleases (Germain et al. 2013; Hirayama 2021). The mechanistic details and regulatory impact of tailing processes on gene expression in plant organelles remain largely unexplored. What is better understood is the role of exonucleases. PNPase and RNase II (also called RNR1) are responsible for generating 3′ ends of transcripts (Stoppel and Meurer 2011; Germain et al. 2013) and are negative regulators of RNA half-lives (Walter et al. 2002; Holec et al. 2006; Hotto et al. 2011; MacIntosh and Castandet 2020). PNPase and RNase II sequentially degrade RNA until they are blocked by a structure or protein (Prikryl et al. 2011; Germain et al. 2012), and the same mechanism occurs in mitochondria (Perrin et al. 2004; Holec et al. 2006). In chlorophytes, PNPase also contributes to RNA stability (Yehudai-Resheff et al. 2007) and also to the generation of some transcripts’ 3′ ends, while other ends are a direct consequence of transcription termination (Rott et al. 1996; Wobbe and Nixon 2013). Chlamydomonas does not have RNase E (Hotto et al. 2020), which might be one indication that different solutions were found in evolution to provide the necessary nucleolytic activities for RNA processing and degradation. With respect to 5′ → 3′ exonuclease activity, only RNase J has been identified in chloroplasts to date. Studies with RNase J mutants have demonstrated that RNase J plays a major role in the removal of noncoding RNA (Sharwood et al. 2011) and in trimming the 5′-end of RNA toward protein blocks in vitro (Pfalz et al. 2009; Luro et al. 2013). In Chlamydomonas, RNase J has only endonucleolytic activity in vitro (Liponska et al. 2018) and it remains to be determined how RNase J fulfills its role in vivo if truly acting only as an endonuclease. No known 5′ → 3′ exonucleases have been found in plant mitochondria (Sharwood et al. 2011) and the presumed ancestral mitochondrial 5′ → 3′ exonuclease, known as Pet127 protein in yeast, has been lost from the plant lineage (Łabędzka-Dmoch et al. 2022). Instead, mitochondrial 5′ ends are formed with the help of a special set of PPR proteins known as “restorer-of-fertility-like” (Rf) proteins (Fujii et al. 2011; Dahan and Mireau 2013; and see the section below on cytoplasmic male sterility [CMS]) that guide unknown endonucleases to sites in UTR regions (Fig. 2, no. 4a; Binder et al. 2016; Fujii et al. 2016; Stoll and Binder 2016; Stoll et al. 2017; Colas des Francs-Small et al. 2018). Importantly, exonucleases require stop signals on RNA in the form of RBPs, as discussed in the next section.

### Rnase companions: helical repeat proteins

#### PPR proteins

The PPR protein family has been reviewed extensively elsewhere (Barkan and Small 2014; Rovira and Smith 2019). Here, we focus on selected aspects of PPR proteins and provide a brief overview of the PPR family. PPR proteins consist of tandem arrays of the eponymous repeat unit, of which different numbers are found per protein, usually organized in tandem. Structural analyses have shown that the repeat array forms a superhelical surface that binds single-stranded RNA with high specificity and affinity (Barkan and Small 2014). This interaction with RNA is sufficiently tight and long-lasting to protect bound RNA against the action of nucleases, which leads to the accumulation of an RNA footprint (Ruwe and Schmitz-Linneweber 2012; Zhelyazkova et al. 2012). Determining RNA footprint abundance can provide clues about the activity of particular PPR proteins. Each PPR unit binds to a single RNA nucleotide (Barkan et al. 2012) via specific hydrogen bonding interactions with 2 key amino acids (Shen et al. 2016). Therefore, decoding the amino acid sequence to predict which RNA sequence a PPR protein binds is possible, at least in some cases (Barkan et al. 2012; Kobayashi et al. 2019). PPR units show some heterogeneity and can be classified into several types based on their repeat composition (Cheng et al. 2016). Generally, PPR proteins are divided into 2 functionally distinct classes: P- and PLS-class PPR proteins (Lurin et al. 2004; Cheng et al. 2016). P-class PPR proteins contain monotypic repeats and are generally associated with tight, passive binding to noncoding RNA, playing a role in RNA stabilization and intron splicing. Binding with high affinity to UTR sequences is necessary to avoid displacement by exonucleases. In the few examples where *K*_D_ values have been measured, recombinant P-class PPRs bind to their cognate RNA in the picomolar–nanomolar range. For example, the equilibrium *K*_D_ values of PPR10 and HCF152 are 10 and 40 pM, respectively (McDermott et al. 2018), leading to stable protein–RNA complexes with lifetimes of 2 h or more. This binding is sufficient to withstand the action of exonucleases and prevent the degradation of bound RNA (Fig. 2; Pfalz et al. 2009; Prikryl et al. 2011). Because PPR proteins bind to most intercistronic spacers, polycistronic RNAs are processed to various lengths depending on where the initial endonucleolytic cleavage occurs, allowing exonucleases to degrade RNA until they are blocked.

PLS-class PPR proteins, which are typically involved in RNA editing (Small et al. 2020; see below), contain a variety of repeat units and C-terminal extensions that are absent in P-class PPR proteins. Several PLS-class PPR proteins have acquired new roles in RNA stabilization, such as CRR2 (Hashimoto et al. 2003; Ruwe et al. 2019), or intron-splicing, such as OTP70 (Chateigner-Boutin et al. 2011), which require long-lasting binding to noncoding RNA. Thus, these proteins fulfill the typical roles of P-class proteins, suggesting that little intrinsic functional difference in RNA binding exists between 2 classes. Indeed, CRR2 generates some of the most abundant RNA footprints in Arabidopsis chloroplasts (Ruwe et al. 2019). Splicing and RNA stability factors linger long enough for the surrounding sequences to be degraded by exonucleases with only RNA footprints remaining, indicating that chloroplasts can dispense with the fraction of PPR proteins that are presumably absorbed by superfluous short RNA footprints. In the case of PPR10, immunoprecipitation assays revealed that this fraction was larger than the amount of PPR10 bound to mature *atpH* or *atpI* mRNA (Ruwe et al. 2016). Further quantification of different RNA isoforms bound by PPR proteins is needed to understand the impact of productive versus nonproductive binding of PPR proteins. Combined with the quantification of PPR proteins, these findings will help determine whether a PPR protein is limiting and potentially regulates the expression of the target RNA.

### Other helical repeat proteins involved in transcript stabilization

The domain organization of PPR proteins, which includes degenerate repeats of helical dimers, is frequently found in nature and such proteins are classified as “helical repeat proteins.” While the PPR protein family is dominant in land plants, helical repeat proteins involved in RNA stabilization and maturation from other subfamilies are found in other plant organelles. PPR proteins are believed to be evolutionarily derived from tetratricopeptide repeat (TPR) proteins, which are known for their propensity to form protein–protein interactions. However, some TPR proteins have evolved to bind RNA, including members of the half-a-tetratricopeptide (HAT) subfamily (Bohne et al. 2016). Moreover, members of the mitochondrial transcription termination factor (mTERF) family (Table 1; Kleine 2012; Macedo-Osorio et al. 2021; Robles and Quesada 2021) can also stabilize RNA (Meteignier et al. 2020). Notably, chlorophyte algae have only a few PPR proteins and instead use related OPR proteins with a slightly longer repeat unit as the main organellar RNA maturation factor. For example, Chlamydomonas has only 14 PPR proteins but over 120 OPR proteins (reviewed in Macedo-Osorio et al. 2021), whereas Arabidopsis has only 1 OPR (Rahire et al. 2012; Kleinknecht et al. 2014; Bohne et al. 2016; Macedo-Osorio et al. 2021). Outside the green lineage, heptatricopeptide repeat (HPR) proteins, which are related to OPR proteins, are found frequently in Apicomplexans, a group of parasitic protists (Hillebrand et al. 2018) and another OPR-like family has been described in a photosynthetic cercozoan (Oberleitner et al. 2020). Helical repeat protein families may have expanded in any particular lineage by chance. Thus, the distinction between HAT, PPR, OPR, and HPR proteins may not be of much functional relevance, particularly since RNA recognition by the different protein families does not appear to differ conceptually. As long as the expansion of helical repeat protein genes is possible, different evolutionary lineages will exploit sequence-specific RNA adaptors to manage their organellar transcripts. This hypothesis could be tested by replacing OPR or HPR proteins with designer PPR proteins in a green alga model species such as Chlamydomonas or in an Apicomplexan model species such as *Toxoplasma gondii*.

### A larger window of opportunity for RNA maturation

The immediate outcome of the action of helical repeat proteins, the most abundant P-class PPR proteins, is the baffling complexity of transcript isoforms, which exceeds bacterial and nuclear transcript heterogeneity. The production of shorter, often monocistronic, forms from longer precursor RNAs has long been assumed to be beneficial for protein production. Chloroplast operons contain genes with very different functions, and separating individual cistrons was speculated to contribute to adjusting individual transcript amounts and improving translation. The results of experiments with in vitro translation systems supported this idea, where the translational output of some mRNAs was strongly influenced by the processing state of the UTRs (Yukawa et al. 2006; Adachi et al. 2012; Kuroda and Sugiura 2014). However, these results did not align with polysome analysis results and more recent ribosome profiling experiments in which all mRNA isoforms, including unspliced mRNA, were translated (Barkan 1988; Zoschke et al. 2013; Zoschke and Barkan 2015; Zoschke and Bock 2018). In addition, precipitation of nascent peptides together with their mRNAs did not show a preference for translation of a particular transcript isoform (Barkan 1988), suggesting that in vivo translation is independent of RNA processing for many, if not most, RNAs. The curious complexity of transcript patterns has drawn attention away from the key effects of helical repeat proteins, including their decisive, direct impact on translation initiation via RNA restructuring (Fig. 2, no. 7) and their impact on transcript half-lives (Fig. 2, no. 5). Although the specifics of the former effect are beyond the scope of this review, the importance of the latter effect is discussed in more detail below.

The half-lives of chloroplast transcripts, determined by blocking transcription and measuring RNA quantities over time, is in the range of hours or even days (Klaff and Gruissem 1991; Kim et al. 1993; Germain et al. 2012; Germain et al. 2013; Szabo et al. 2020). Noninvasive in vivo labeling methods reported somewhat shorter but comparable half-lives (Szabo et al. 2020). These values are in stark contrast to those of bacteria, where mRNA half-lives were generally measured in minutes (Selinger et al. 2003; Kristoffersen et al. 2012). Helical repeat proteins are responsible for the elongated half-lives for many RNAs. The deletion of a stabilizing helical repeat protein often results in the apparent absence of any remaining RNA, that is, its half-life approaches zero. This has 2 implications: first, no other stabilizing factors can compensate for the action of helical repeat proteins; and second, no window of stability exists after transcription since decay occurs instantaneously. This concurred with the findings that many PPR proteins and RBPs are associated with nucleoids and the location of transcription (Pfalz et al. 2006; Majeran et al. 2011), and are thus available to protect nascent transcripts.

RNA stabilization by helical repeat proteins opens a window of opportunity that facilitates slow RNA maturation steps (Fig. 2). Among them, RNA splicing requires folding of the catalytic core of ribozymatic group II introns and is a comparatively slow process, given that many RNA–RNA and protein–RNA interactions are needed to achieve the correct RNA-fold. Chloroplast splicing is particularly inefficient and slow, as indicated by the substantial amounts of unspliced mRNAs that accumulate under normal growth conditions (see for example RNA gel blots on wild-type samples in Jenkins et al. 1997). Helical repeat proteins extend the half-lives of intron-containing RNA precursors, thus supporting intron folding and splicing (Fig. 2, no. 6). For example, 2 PPR proteins positively affect the transcript stability of the 2 *rps12* mRNA halves, supporting the *trans*-splicing of *rps12* (Lee et al. 2019). The same holds true for *psaA trans*-splicing factors in Chlamydomonas (reviewed in Kück and Schmitt 2021). PPR5, another PPR protein that supports splicing by increasing transcript stability, is required for splicing an intron in the *trnG*-UCC primary transcript (Beick et al. 2008). PPR5 binds to an endonuclease-sensitive site, which likely blocks access by RNase. Furthermore, the chloroplast PPR protein SOT1 (also named PPR53 in maize) facilitates the splicing of *ndhA* by increasing the transcript stability of *ndhA* through association with CAF2 (Li et al. 2021). Mathematical modeling of splicing efficiency under different RNA stability conditions demonstrates that low turnover rates favor the accumulation of spliced versus unspliced transcript isoforms (Fig. 2, nos 8/9). Similar effects are observed for edited and unedited mRNA (Fig. 2, no. 10). Modulating RNA stability can thus dramatically alter the efficiency of organellar RNA maturation. Of course, extending the time for splicing might go hand in hand with the direct function of helical repeat proteins in RNA folding, and thus the formation of catalytic centers for group II introns (Fig. 2, no. 4b). RNA splicing and participating factors are explored in greater detail in the next section.

### Organellar splicing machinery

Plant organelles predominantly contain Group II introns, whereas Group I introns are prevalent in algae such as Chlamydomonas. Group I and II introns are ribozymes that catalyze their own splicing, necessitating complex RNA–RNA interactions (Mukhopadhyay and Hausner 2021). Plant organellar introns have substantially diverged from the canonical intron structure during evolution, while splicing chemistry has been mostly conserved (Hausner et al. 2006). Proteinaceous cofactors are required to obtain a catalytically active intron structure in vivo. The following sections introduce some of these cofactor proteins, focusing on select nuclear-encoded RBPs.

### Helical repeat proteins in splicing

Among the nuclear-encoded helical repeat proteins required for RNA splicing, many belong to the PPR protein family and a few belong to the mTERF family (Hammani and Barkan 2014; Lee et al. 2021). More than 60 (mostly P-class) PPR proteins play a proposed role in the splicing of different organellar introns (Supplemental Table S2). In most cases, these factors have been determined purely by genetic means, which creates bias. For example, although splicing factors have been identified for most introns in mitochondrial *nad* genes, very few have been identified in mitochondrial genes encoding subunits of other respiratory complexes or ribosomes, presumably because the loss of such splicing factors would be lethal. Furthermore, comparing accumulated unspliced versus spliced mRNA in mutants is not a failsafe method to identify splicing factors since accelerated RNA turnover inevitably leads to an increased ratio of unspliced to spliced RNA (Fig. 2, no. 9). Thus, RNA stabilization factors can be misclassified as splicing factors. Only select cases have identified an interaction between a putative splicing factor and its target intron, as demonstrated by coimmunoprecipitation techniques (e.g. Beick et al. 2008; Khrouchtchova et al. 2012; Lee et al. 2019; Hammani and Barkan 2014) or gel shift experiments (e.g. Aryamanesh et al. 2017; Ito et al. 2018). Thus, mechanistic insights come from only a few case studies. A particularly complex case is the first intron of *rps12* mRNA, which is found divided between 2 chloroplast loci, that is, it is *trans*-spliced. In vivo and in vitro RNA-binding studies supported by footprint analyses and code-based target site predictions have demonstrated that 2 PPR proteins bind close to the free ends of the 2 intron halves (Schmitz-Linneweber et al. 2006; Aryamanesh et al. 2017; Tadini et al. 2018; Lee et al. 2019). Binding of PPR proteins maintains the target sites in a single-stranded state (Prikryl et al. 2011), which may be necessary to form the correct structure for splicing (Fig. 2, no. 4b).

The *trans-*splicing of chloroplast *psaA* mRNA in Chlamydomonas is an even more complex splicing event. *psaA* mRNA is assembled from 4 independently generated transcripts (Goldschmidt-Clermont et al. 1991). Initial genetic analyses revealed that at least 14 nuclear loci are involved in the maturation of this single chloroplast mRNA. To date, genes for half of these loci have been identified (Kück and Schmitt 2021). Among them, *RAA1*, *RAA8*, and *RAT2* encode proteins that have OPR domains (Balczun et al. 2005; Merendino et al. 2006; Marx et al. 2015). Together with other factors, they form 2 major subcomplexes, excising either introns *i1* or *i2*. These subcomplexes most likely interact to coordinate the splicing process (Lefebvre-Legendre et al. 2016; Kück and Schmitt 2021), thereby forming a large splicing machine. Therefore, *psaA trans-*splicing has often been interpreted as an example of how nuclear spliceosomes could have evolved from Group II introns.

### CRM proteins: splicing specialists and the question of splicing complexes

While PPR proteins are the jack of all trades in organellar RNA maturation, including splicing, the chloroplast RNA splicing and ribosome maturation (CRM) family of RBPs is more specialized, with all but 1 member involved in RNA splicing in chloroplasts and mitochondria (Wang et al. 2022). The best-characterized CRM family member is CRS1, which is essential for splicing the *atpF* intron (Till et al. 2001). In vitro RNA structure analysis in the presence and absence of CRS1 indicated that CRS1 promoted intron folding via specific interactions with 2 intron domains (Ostersetzer et al. 2005). Whether intron remodeling is a general feature of members of the CRM family remains unclear, but RNA chaperone activity has been demonstrated for at least one CRM protein, CFM4, by using CFM4 to complement a chaperone-deficient *Escherichia coli* mutant (Lee et al. 2014).

CRM proteins have been shown to interact directly with CRM relatives, PPR proteins, and other splicing factors in chloroplasts (Ostheimer et al. 2003, 2005; reviewed in Wang et al. 2022) and mitochondria using one-on-one interaction studies (Chen et al. 2019; Zhao et al. 2020; Wang et al. 2020b; Fan et al. 2021; Cao et al. 2022). Whether these splicing factors form larger complexes, in the manner of a spliceosome, remains largely unclear. CRM proteins may attach to introns independently and/or consecutively. The complex formation of multiple splicing factors has been ascertained for *trans*-splicing (Kück and Schmitt 2021); genetic data coupled with RNA coimmunoprecipitation suggest that organellar introns are usually served by at least 2 splicing factors and often by many more (Germain et al. 2013; Lee et al. 2019); Biochemical experiments have shown that chloroplast introns are found in large ribonucleoprotein particles that include multiple different splicing factors (eg, Kroeger et al. 2009; Kück and Schmitt 2021). However, some splicing factors may act consecutively rather than simultaneously. In the case of chloroplast *ycf3* intron 1, mutants of six PPR proteins, three CRM proteins, and CRS2 showed reduced splicing efficiency (Supplemental Table S2) (Jenkins et al. 1997; Ostheimer et al. 2003). A similar number of factors were reported for the chloroplast *ndhA* intron (Supplemental Table S2). However, many proteins that have been genetically linked to organellar splicing defects have not been shown to be associated with the affected intron, leaving open the possibility that these splicing defects are indirect effects. All of the mutants described herein induced more or less severe defects in plant and chloroplast development, including albinism and other pigment deficiencies, as well as retarded or aberrant leaf growth. Such developmental defects may very well affect splicing indirectly. Retrograde signals from defective chloroplasts to the nucleus are known to affect a multitude of genes encoding proteins involved in chloroplast functions, possibly including splicing factors. Other effects could be based on physiological changes in defective chloroplasts. Indeed, reduced or lost photosynthetic capacity likely impacts ion homoeostasis in chloroplasts, which can affect RNA folding and thus intron splicing. This idea is supported by recent findings in which changes in potassium levels in chloroplasts affected rRNA maturation, possibly via RNA folding (Schock et al. 2000; DeTar et al. 2021). Notably, magnesium is a potent mediator of Group II intron splicing in mitochondria (Schock et al. 2000; Gregan et al. 2001). Thus, ion concentration changes in defective organelles could lead to secondary splicing defects. Additional factors likely contribute to the efficiency of intron removal, and individual introns are likely to be affected nonuniformly by such changes given different intron structures. For example, *ycf3* intron 1 and the *ndhA* intron are particularly sensitive to chloroplast deficiencies. Additionally, *nad2* intron 1 is similarly sensitive in mitochondria, and unspliced RNA containing this intron accumulated in many different mutants (Colas des Francs-Small et al. 2014). Unraveling the individual splicing kinetics for various Group II introns in organelles and elucidating why different sets of proteins are needed for different introns will be exciting. Nevertheless, carefully assessing the function of any presumed splicing factor in intron removal remains important. Data showing interactions between factors and introns in vivo are needed to support the presence of direct effects. In addition, control mutants with similar but mechanistically unrelated chloroplast developmental defects will help discern direct from indirect effects.

### Honorary mentions

In addition to helical repeat proteins, a number of smaller protein families are involved in RNA splicing, such as plant organelle RNA recognition (PORR) proteins or the DUF794 protein family (Kroeger et al. 2009; Watkins et al. 2011). Moreover, a number of orphan splicing factors, such as the RanBP2-type zinc finger protein OZ2 (Bentolila et al. 2021) and the curious DNA- and RNA-associated Whirly1 protein, are involved in RNA splicing (Prikryl et al. 2008; Melonek et al. 2010; Krupinska et al. 2022). Given that Group II introns are complex RNA structures that require multiple RNA–RNA interactions and convoluted RNA-folding pathways during and after synthesis, it is unsurprising that several RNA helicases capable of removing misfolded RNA structures have been identified as splicing factors in plant organelles (Kohler et al. 2010; Asakura et al. 2012; He et al. 2012; Gu et al. 2014; Carlotto et al. 2016; Bobik et al. 2017). For all helicases, the intron structures targeted by unwinding activity remain to be determined. Further, ribosomal proteins are an unexpected group of proteins that were recently added to the ever-growing list of plant organellar splicing factors (Wang et al. 2020a). Specifically, members of the nuclear-encoded uL18 ribosomal protein family are targeted to either plant mitochondria or chloroplasts, supporting the splicing of mitochondrial or chloroplast introns, respectively (Wang et al. 2020a). While 2 out of 8 uL18 proteins were identified as organelle ribosomes, 2 others cosedimented with introns, thus promoting splicing in gradient centrifugation assays. However, the prevalence of this functional change in ribosomal proteins remains unknown.

In summary, the diversity of RNA-binding domains that are active during splicing is impressive. Splicing factors are recruited from canonical RBP families and also opportunistically recruited from other sources. For example, CRS2 is derived from a peptidyl-tRNA hydrolase (Jenkins and Barkan 2001) and RNC1 harbors remnants of the RNase III domains (Watkins et al. 2007). Both factors have lost their original enzymatic activity. In addition, factors important for DNA metabolism and ribosomal proteins have been neo-functionalized to become splicing factors, such (WHY1, uL18 members; Prikryl et al. 2008; Wang et al. 2020a). Overall, eukaryotic cells invest in a plethora of factors to splice organellar RNA.

### Outlook: in vitro splicing

Recently, a chloroplast-derived in vitro system was presented that enables detailed analysis of the splicing process and unraveling of mechanistic details (Inaba-Hasegawa et al. 2021). This indicated that chloroplast-derived introns underlie the expected mechanism. However, deviations were also observed. For example, the exon-binding site 2–intron-binding site 2 interaction did not appear to be essential for the splicing process. In addition, the first nucleotide of the 3′ exon was shown to be irrelevant in splicing. Moreover, the conditions for splicing in chloroplasts were relatively relaxed compared to those in bacteria. Expansion of the proteinogenic machinery for chloroplast introns may play an important role here. The greater number of *trans*-acting factors supporting the structural formation of Group II introns allows greater variability in intron sequences, which is analogous to the evolution of the spliceosome as a *trans*-splicing machine, requiring very few and degenerate target sequences in introns to carry out its function.

### And tRNA maturation

As they are derived from prokaryotes, chloroplasts and mitochondria possess bacterial-like translation machinery. However, while chloroplast ribosomes strongly resemble those of bacteria (Bieri et al. 2017; Perez Boerema et al. 2018), plant mitoribosomes have diverged significantly during evolution. In particular, their rRNA structure and protein composition differ extensively from those of *E. coli* ribosomes (Bonen 2004; Waltz et al. 2021, 2019). Moreover, most plastids encode a complete set of tRNAs sufficient to decode the genetic code of the plastome (Alkatib et al. 2012; Cognat et al. 2013), whereas plant mitochondrial genomes encode only 0 to 40 tRNA genes and depend on the import of the remaining components from the cytosol (Mower 2020; Warren and Sloan 2020). rRNAs contained in organellar ribosomes are usually encoded in one operon. In Arabidopsis chloroplasts, a 7.4-knt polycistronic precursor transcript encodes the 16S rRNA, 2 Group II intron-containing tRNAs, as well as the 23S, 4.5S, and 5S rRNAs. The precursor is then processed at multiple levels to produce mature tRNA and rRNA, similar to what occurs in bacteria where many factors are involved in the biogenesis of ribosomes, including rRNA maturation. In recent years, several factors implicated in 16S and/or 23S rRNA maturation have been discovered and functionally characterized based on mutant phenotypes (Germain et al. 2013). Many of these factors are RNases (Stoppel and Meurer 2011; Hotto et al. 2015; Liu et al. 2015). However, secondary effects on chloroplast rRNA maturation in mutant lines could not be excluded, and only a few defined binding sites (BS) on rRNA precursors have been mapped. For example, the PPR-SMR protein SOT1 in Arabidopsis, similar to its ortholog PPR53 in maize, binds to the 5´ end of the dicistronic 23S–4.5S rRNA precursor (Wu et al. 2016; Zoschke et al. 2016), as indicated by in vitro RNA-binding assays and the absence of a corresponding RNA footprint in a *sot1* mutant (Wu et al. 2016). In addition to protecting the 5′ end of the precursor RNA, SOT1 may have endonucleolytic activity that mediates the maturation of the rRNA precursor transcript (Zhou et al. 2017). Other maturation factors that recognize the 23S rRNA precursor include the RBD1 and the DEAD-box RNA helicase RH50 (Wang et al. 2016; Paieri et al. 2018). 16S rRNA maturation depends on the factors RBF1 and RAP, with the latter representing the sole OPR protein in Arabidopsis (Fristedt et al. 2014; Kleinknecht et al. 2014). RAP binds to the 5′-region of the 16S rRNA precursor and assists in maturation. Strikingly, RAP, similar to the rRNA maturation-related chloroplast GTPases DER and RBD1, displays nucleoid localization, which supports the idea that rRNA maturation and ribosome assembly occur close to the chloroplast genome (Jeon et al. 2014; Kleinknecht et al. 2014; Wang et al. 2016). Indeed, many ribosome biogenesis factors that function in rRNA maturation and ribosome assembly have been identified in the proteomic analysis of chloroplast nucleoids from maize (Majeran et al. 2011; Germain et al. 2013). As discussed previously (Bohne 2014), nucleoids might provide a scaffold for the establishment of an intraorganellar microenvironment, facilitating ribosome assembly via substrate channeling and avoiding precocious association of mRNA with immature 30S ribosomal subunits.

Similar to rRNA maturation, the maturation of organellar tRNA precursor transcripts is generally similar to that in bacterial counterparts, although some deviations have been reported (Frank and Pace 1998; Rossmanith 2012; Salinas-Giegé et al. 2015). One example is RNase P, an enzyme that removes the 5´-region of organellar tRNA precursors. In bacteria, RNase P is usually a ribonucleoprotein that contains a catalytically active RNA moiety. However, an RNase P was identified in organelles that is composed only of protein, named proteinaceous RNase P (PRORP). PRORP belongs to a class of PIN-like ribonucleases that contain an additional PPR domain (Gobert et al. 2010, 2019; Bhatta and Hillen 2022). Although organellar PRORPs are active as single-subunit enzymes in vitro, recent interactome studies have revealed that they can also form high-molecular-weight complexes containing ribosomes and other RNA maturation enzymes (Zhou et al. 2015; Bouchoucha et al. 2019).

The endonuclease RNase Z is involved in the maturation of the 3′-end of tRNA. Genetic analysis has revealed that the plastid enzyme is essential for chloroplast biogenesis in rice (Long et al. 2013). Interestingly, the dually located mitochondrial/chloroplast mTERF6 protein from Arabidopsis has been shown to be specifically involved in the maturation of plastid tRNA^Ile^ (Romani et al. 2015). This raises the possibility that individual tRNA maturation steps during the general steps of tRNA maturation might enable fine-tuning of balanced tRNA accumulation in organelles.

### RNA editing

RNA transcripts in plant organelles undergo many base modifications that alter base-pairing and thus RNA folding and translation. These include many different modifications to tRNAs and rRNAs, but also modification of mRNAs. Adenosine methylation (m^6^A) of mRNAs is common in mitochondria and chloroplasts, although the functional impact of this is not yet clear (Wang et al. 2017; Murik et al. 2020). Of much more interest to researchers has been the deamination of cytidine to uridine, commonly referred to as RNA editing (Small et al. 2020), as this changes the sequence of the mRNA and very often results in a change in the sequence of the encoded protein. C-to-U RNA editing is nearly ubiquitous in land plant organelles, lacking only from marchantiid liverworts (Groth-Malonek et al. 2007; Rüdinger et al. 2008), but occurs at greatly different frequencies in different plants, with, for example, only 2 (Miyata and Sugita 2004) and 11 (Rüdinger et al. 2009) edited sites in *Physcomitrium patens* chloroplasts and mitochondria, respectively, but up to several thousand in each organelle in some *Selaginella* species (Hecht et al. 2011; Oldenkott et al. 2014; Smith 2020; Zhang et al. 2022). In angiosperms, commonly a few dozen cytidines are edited in chloroplasts (Giegé and Brennicke 1999; Chateigner-Boutin and Small 2007; Ruwe et al. 2013; Sloan et al. 2018) but several hundred in mitochondria (Giegé and Brennicke 1999; Chateigner-Boutin and Small 2007; Ruwe et al. 2013; Sloan et al. 2018). The hydrolytic deamination of cytidine to uridine is usually considered irreversible under physiological conditions, as discussed in Gerke et al. (2020), but remarkably “reverse” editing (U-to-C) does occur in some hornworts (Kugita et al. 2003), lycophytes (Grewe et al. 2011), and ferns (Hiesel et al. 1994); and can even be the dominant form of editing in some species (Knie et al. 2016; Gerke et al. 2020). RNA editing has attracted much research since its discovery and the historical development of the field has been reviewed recently (Small et al. 2020), so here we will confine ourselves to describing our current understanding of the editing machinery and how RNA editing is integrated with other steps in plant organellar RNA maturation.

**Figure 3.**
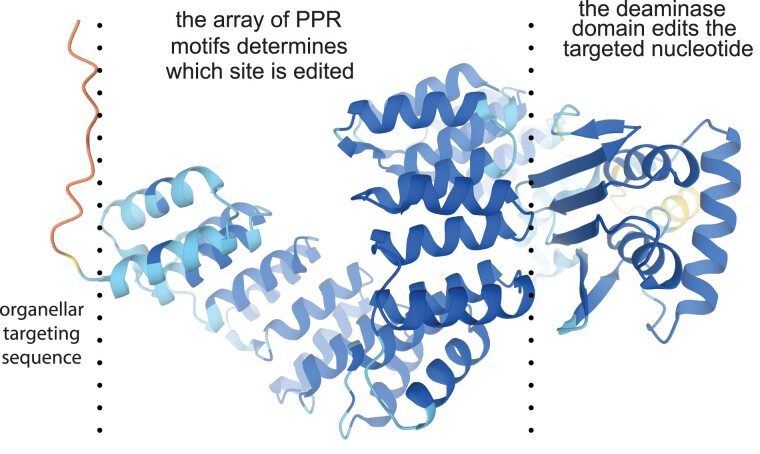
Structure of a DYW–PPR protein. PPR proteins are generally characterized by their extended tract of helical elements that is flanked at its N-terminus by an unstructured targeting peptide. While the PPR tract is responsible for base-specific RNA recognition of all PPR proteins, DYW–PPR proteins have an additional, C-terminal DYW domain with base deamination activity. The structure shown is of MEF1 (Zehrmann et al. 2009) as predicted by AlphaFold (Jumper et al. 2021).

The principal factors involved in RNA editing are PLS-class PPR proteins (Yagi et al. 2013b; Cheng et al. 2016). These act as specificity factors, determining which cytidines are edited (Okuda et al. 2006). The PPR array in these proteins binds to the RNA 5′ of the site to be edited, with the last motif of the PPR array aligned with the nucleotide at position −4 (Barkan et al. 2012; Yagi et al. 2013a). PPR editing factors have a C-terminal domain comprising 2 PPR-like helix-turn-helix motifs (named E1 and E2) and a cytidine deaminase domain of about 160 amino acids, usually terminating in a characteristic Asp–Tyr–Trp triplet that has led to this domain being commonly referred to as the “DYW” domain (Fig. 3; Lurin et al. 2004; Cheng et al. 2016). The DYW domain contains a typical cytidine deaminase active site (Salone et al. 2007; Wagoner et al. 2015) with a zinc ion (Hayes et al. 2013; Boussardon et al. 2014) bound by an HxE (x)_n_CxxC motif which based on similarity to other deaminases (Iyer et al. 2011), probably acts in concert with an essential glutamate residue (Hayes et al. 2015) to catalyze cytidine deamination. Crystal structures of the deaminase domain suggest that the active site may be occluded by a “gating domain” (Takenaka et al. 2021), implying a conformational change in binding to the target site that allows access to the catalytic center. The precise placing of the deaminase domain on the RNA by the PPR array leads to highly specific base editing; in most cases (but not all), cytidines adjacent to the editing site are not edited (Choury et al. 2004; Arenas-M et al. 2013). In chloroplasts, most editing factors only edit a single site in the transcriptome, whereas in mitochondria, editing of multiple sites is more common. Where multiple sites are edited, the upstream sequences that form the PPR binding site are similar enough for the same PPR array to bind them both/all (Hammani et al. 2009).

The factors responsible for “reverse” editing appear to be a subclade of DYW proteins whose presence and abundance correlate strongly with U-to-C events (Gutmann et al. 2020), and whose PPR arrays are a good match for U-to-C editing sites (Gerke et al. 2020). Synthetic proteins based on these sequences can catalyze U-to-C editing (Ichinose et al. 2022). How 2 very similar sets of proteins catalyze reactions in opposite directions will be an intriguing puzzle to solve.

Some PPR editing factors are “broken” into 2 parts, one providing the PPR array, and the other the deaminase domain (Boussardon et al. 2012; Gutmann et al. 2017). In some cases, these interactions are highly specific (e.g. CRR4 and DYW1 in Arabidopsis; Boussardon et al. 2012), but in others a single DYW domain may provide deaminase activity to many different PPR partners, as appears to be the case for DYW2 (Andrés-Colás et al. 2017; Guillaumot et al. 2017; Malbert et al. 2020) and MEF8 (Verbitskiy et al. 2012; Diaz et al. 2017; Yang et al. 2022) in Arabidopsis and PCW1 in maize (Wang et al. 2022). Thus, although the number of PLS-class proteins in the proteome correlates fairly well with the number of editing events in the organelle transcriptome (Fujii and Small 2011; Gutmann et al. 2020), the number of DYW proteins may not.

Single PPR–DYW proteins contain everything needed to catalyze the editing of their target RNA, as demonstrated by the reconstitution of C-to-U editing in bacteria (Oldenkott et al. 2019; Bernath-Levin et al. 2021; Royan et al. 2021; Ichinose et al. 2022), mammalian cells (Ichinose et al. 2022; Lesch et al. 2022), and in vitro (Hayes and Santibanez 2020). However, in plant organelles, numerous other “editing factors” have been identified by genetic approaches or protein–protein interaction screens using PPR proteins as bait (Sun et al. 2016). The best-known of these are the RIP or MORF proteins (Bentolila et al. 2012, 2013; Takenaka et al. 2012) encoded by a small multigene family. All of these appear to be targeted to mitochondria or chloroplasts, where they interact with PPR editing factors (Takenaka et al. 2012; Härtel et al. 2013b; Brehme et al. 2015; Glass et al. 2015; Bayer-Császár et al. 2017; Ren et al. 2020) and influence their RNA-binding activity (Yan et al. 2017; Royan et al. 2021). RIP/MORF mutants generally show multiple (overlapping) editing defects, suggesting each RIP/MORF protein can interact with many different PPR proteins, and that many of these PPR proteins are at least partially reliant on their assistance for full activity (Takenaka et al. 2012; Bentolila et al. 2013). Many other editing-associated factors have been identified, including RBPs such as RRM-domain proteins (Tillich et al. 2009a; Sun et al. 2013; Shi et al. 2015; Shi et al. 2017; Searing et al. 2020) and the zinc-finger protein OZ1 (Sun et al. 2015; Gipson et al. 2022), the RNA helicase ISE2 (Bobik et al. 2017), and P-class PPR proteins such as NUWA (Andrés-Colás et al. 2017; Guillaumot et al. 2017). The role of these auxiliary proteins in RNA editing is not entirely clear. Often the same or related proteins are implicated in other RNA maturation activities, suggesting that they may not be tightly or solely associated with the editing machinery. The concept of an “editosome” has been advanced in the literature (Sun et al. 2016) but the experimental evidence for this hypothetical multi-subunit complex remains inconclusive, with the complexes detected so far being extremely heterogeneous in size and difficult to purify intact (Bentolila et al. 2012; Huang et al. 2019; Sandoval et al. 2019). Given the demonstration that a single editing factor alone can catalyze editing on a specific target (Hayes and Santibanez 2020), it seems possible to us that auxiliary editing factors act in series via weak transient interactions to facilitate editing rather than forming a stable, well-defined complex.

### Editing in relation to other steps in RNA maturation

The kinetics of RNA editing are not yet well-understood, but it appears to be a relatively fast process, given that editing at most sites generally exceeds 90% (Ruwe et al. 2013). In comparison, splicing tends to be much slower, with a greater proportion of unprocessed transcripts present in the organelle. Thus editing often precedes splicing, and indeed, in some cases splicing is dependent upon prior editing, either because the editing creates a sequence motif required for the intron-splicing mechanism (Castandet et al. 2010; Xu et al. 2020), or because binding of the PPR array to the RNA is needed for intron folding (Yap et al. 2015).

It is obviously preferable that editing precedes translation, given that should unedited or partially edited transcripts be translated, proteins with the “wrong” amino acid sequence would be generated. Such proteins can be deleterious (Hernould et al. 1993). How this is avoided is not yet clear. Partially edited mRNAs do not appear to be excluded from ribosomes (reviewed in Zoschke and Bock 2018), and in mutants lacking specific editing factors, the unedited transcripts are translated normally, giving rise to defective proteins. Yet in wild-type plants there is no evidence of any complete translation products being produced from unedited or partially edited transcripts (Lu and Hanson 1994). Either any such products are rapidly degraded, or the editing factors themselves prevent complete translation of unedited mRNA by remaining bound until editing is complete. However, where it has been tested, PPR editing factors bind almost equally tightly to edited or unedited target RNA (Okuda et al. 2014). The situation is particularly acute in hyper-editing plants such as ferns and lycophytes where thousands of editing factors edit thousands of sites and translation must surely be deferred until editing is complete. One way in which this is achieved is by the mRNA being untranslatable until edited because it lacks a start codon (Hirose and Sugiura 1997; Chotewutmontri and Barkan 2016) or contains premature stop codons (Kugita et al. 2003). Start codons can be introduced by C-to-U editing of an ACG codon, and U-to-C editing can alter UAA, UAG and UGA stop codons to CAA (Gln), CAG (Gln), or CGA (Arg), respectively. Such start and stop codon editing is highly prevalent in hyper-editing plants (Kugita et al. 2003; Grewe et al. 2011; Li et al. 2018). As long as these translation-determining editing events occur late in the process, translation of unedited mRNAs can be avoided. Indeed, long-read single-molecule sequencing has recently shown for the Arabidopsis *ndhD* mRNA that the last of 4 editing sites to be processed within this transcript is within the start codon (Guilcher et al. 2021).

### CMS and nuclear factors involved in fertility restoration

CMS is a fertility phenotype in plants determined by the expression of aberrant mitochondrial genes. The CMS trait becomes visible only during flowering when otherwise healthy-looking plants fail to produce functional pollen (Chase 2007). Genes that determine CMS are created during recombination events in mitochondrial genomes and often display a chimeric structure, that is, they are composed of conserved mitochondrial sequences as well as unique sequences of unknown origin (reviewed in Chen and Liu 2014). Newly created CMS genes can be expressed via fragments of mitochondrial promoter sequences or through cotranscription with upstream mitochondrial genes (Hanson and Bentolila 2004; Tang et al. 2017).

The presence of CMS-associated genes in the mitochondrial genome is often masked by nuclear *restorer-of-fertility* (*Rf*) genes, which block their expression and restore pollen production and plant fertility by reducing their deleterious effects (Hanson and Bentolila 2004). As a result, CMS-associated genes are often detected only by interspecific crosses or somatic cell fusions that create alloplasmic plants carrying the cytoplasm from 1 plant species and nucleus from a different species (Chase 2007). Several *Rf* genes have been cloned, the majority of which belong to a specific clade in the PPR family, referred to as *Rf-like* (*RFL*) genes (Fujii et al. 2011; Dahan and Mireau 2013; Gaborieau et al. 2016). Molecular studies have indicated that Rf-PPR proteins have at least 2 modes of action. In the first mode of action, some Rf proteins bind and induce cleavage of CMS-causing transcripts (Menassa et al. 1999; Wang et al. 2006; Huang et al. 2015; Liu et al. 2016; Melonek et al. 2021). However, the mechanism by which Rf proteins induce RNA cleavage remains unclear. Rf proteins do not contain any known endonuclease motifs, and cleavage generally occurs up to 100 nt 3′ of the Rf-binding site, making it unlikely that the Rf protein itself is involved (Colas des Francs-Small et al. 2018). PRORP reportedly performs the RFL2-promoted cleavage of *orf29* in Arabidopsis (Fujii et al. 2016), but the endonucleolytic activity remains elusive in other cases (Colas des Francs-Small et al. 2018). The second mode of action was documented in molecular studies of the Ogura CMS system in rapeseed (Wang et al. 2021). The *Rfo*/*PPR-B* protein was demonstrated to suppress the expression of the CMS transcript (*orf138*) by impeding its translation via ribosome blockage rather than by inducing its cleavage (Wang et al. 2021).

Recently, several *Rf* gene candidates in rye and barley were found to encode members of the mTERF family (Bernhard et al. 2019; Vendelbo et al. 2021). No sequence similarity exists between mTERF and PPR proteins; however, similar to PPRs, mTERFs consist of α-helical repeats and function in RNA-associated processes (Meteignier et al. 2020). Genome-wide studies of mTERF proteins in plants have shown that a group of *mTERF* genes shares several genomic features with the RFL-PPR clade (Walkowiak et al. 2020; Melonek and Small 2022). Similar to RFL-PPRs, 1 clade of *mTERF* genes is highly expanded in plants, especially in cereals (Walkowiak et al. 2020; Melonek and Small 2022), and is organized into clusters adjacent to or intermixed with RFL-PPR clusters in the genome (Melonek and Small 2022). The location of RFL-mTERF clusters overlaps with the mapped *Rf* loci in wheat and rye (Walkowiak et al. 2020; Melonek and Small 2022). Based on these discoveries and the analogous relationship with RFL-PPRs, this clade was named RFL-mTERF (Walkowiak et al. 2020). Nevertheless, the contribution of RFL-mTERF proteins to fertility restoration in plants remains to be elucidated.

### Why are there so many RBPs in plant organellar RNA maturation?

#### Complexity gain without selective advantages?

**Figure 4.**
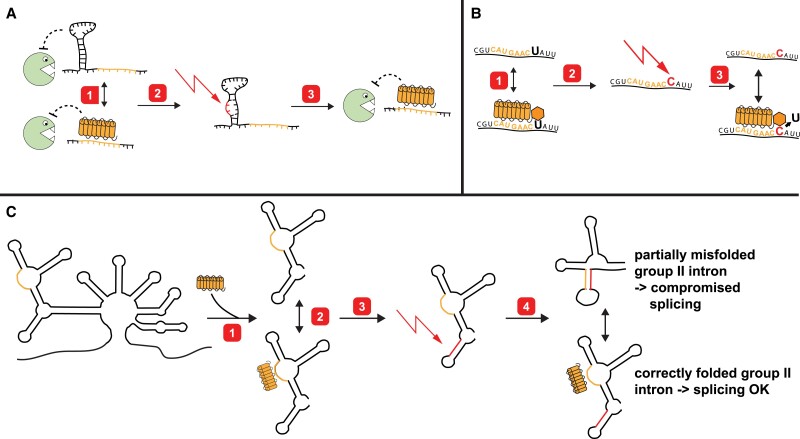
Examples for the evolution of RNA:protein interactions according to the CNE theory. **A)** Suppression of a loss of an important RNA secondary structure by a helical repeat protein. The 5′-terminus of an mRNA is protected against exonucleolytic degradation by RNase J via a stem-loop. 1, A helical repeat protein that can bind in the vicinity of the stem-loop (binding site marked in orange) emerges in evolution. The helical repeat protein can serve as an alternative to the stem-loop to protect the mRNA against 5′-to-3′ degradation. 2, A mutation destroys the stem-loop (marked in red). 3, This mutation is, however, neutral in the background of the helical repeat protein stabilizing the RNA independently. **B)** Suppression of a T-to-C mutation by RNA editing. A coding sequence requires a uridine (large U) at a particular site to encode the correct amino acid and thus keep the corresponding protein functional. 1, A DYW–PPR protein (hexagon = DWY-domain) emerges in evolution that can bind upstream of the critical U (binding site in orange). 2, Whether or not the DYW–PPR protein binds to the target sequence is at first irrelevant—all messages encode the correct protein. A T-to-C mutation at the DNA-level leads to a codon change that is detrimental to the corresponding protein. 3, The mutation is however neutral in the background of the DYW–PPR protein that performs C-to-U base deamination at the RNA level and thus restores the correct codon. **C)** Suppression of a mutation negatively affecting the structure of a group II intron. A group II intron requires folding into the correct structure (a simplified 2D-representation is shown) to allow forming of the ribozyme catalytic center and thus intron removal. 1, A helical repeat protein emerges in evolution that can bind to a single-stranded region of the intron (binding site shown in orange). 2, Whether interaction with this helical repeat protein occurs or not is in the beginning irrelevant for splicing. 3, A mutation leads to a sequence element that is prone to base-pair with the sequence marked in orange. 4, Base-pairing between the mutated (red) sequence and the orange sequence leads to an unproductive alternative intron structure that blocks splicing. However, binding of the helical repeat protein forces the orange sequence element into single-strandedness, preventing detrimental misfolding, and fostering continuous splicing.

The immediate gut response to complexity in biological systems is that the intricacies observed serve a purpose, for example in making a process more efficient or providing regulatory means for a process. Given that many RBPs are essential for chloroplast or even plant development, the notion of importance equaling evolutionary advantage seems even more logical. However, there is no evidence that the vast majority of RBPs serve a regulatory purpose or improve the efficiency of existing processes. Other evolutionary theories have been proposed to explain the complexity of plant organellar RNA maturation based on the peculiarity of the organellar genome in the context of the nuclear host genome (Maier et al. 2008; Gray et al. 2010; Lukes et al. 2011; Castandet and Araya 2012). These hypotheses begin with the idea that organellar genomes can accumulate deleterious mutations, but that these mutations fail to be disadvantageous if a nuclear factor suppresses their effects. The most thorough elaboration of this idea is the constructive neutral evolution (CNE) theory (Stoltzfus 1999; Lukes et al. 2011). Although only a general theory, CNE is particularly relevant for DNA-containing endosymbiotic organelles, suggesting that neutral protein–protein, RNA–RNA, or protein–RNA interactions occur among host factors and organellar factors. Among these interactions, the latter are the most relevant for our considerations. While being neutral at the start, in the case of a deleterious mutation in a particular host factor, a corresponding organellar factor might ensure the neutrality of the mutation. Consider the following tangible examples in the context of organelle biology: (i) transcript stability versus exonucleases can be mediated via terminal stem loops. If a helical repeat protein can potentially recognize a sequence element at such a terminus, the stem loop can be lost, with no effect on stability (Fig. 4A). (ii) A T → C mutation in a reading frame leading to a deleterious amino acid shift or even a stop codon can be suppressed in the presence of a PLS–PPR protein leading to a C → U editing event at this site (Fig. 4B). (iii) RNA–RNA interactions important for intron catalysis can be compromised if mutations allow alternative, energetically favored interactions. RBPs can resolve such misfolding by acting as RNA chaperones or as helical repeat proteins to maintain certain sequences in a single-stranded conformation (Fig. 4C).

If the CNE holds true, a multitude of neutral interactions should be observable. While neutrality is impossible to ascertain since not all conditions under which an interaction might become relevant can be analyzed, a number of examples might fall into this neutral class of host–organelle interactions. For example, PPR proteins can interact with target RNA without having any particular effect on the RNA transcript level. A case in point is the chloroplast PPR protein CRR2, which produces RNA footprints without showing any RNA aberrations in *crr2* null mutants, such as in the *matK* reading frame. Among the targets of CCR2, several antisense RNA species, including *ycf2*, *rpoC2*, and *rpl23*, do not seem to serve any purpose, nor do they affect the corresponding sense RNA (Ruwe et al. 2019). The expression of all 4 targets is essential for chloroplast and plant survival, but *crr2* mutations have none of the expected macroscopic phenotypes that would occur if any of these 4 genes showed reduced expression. The only effect observed after *crr2* knockout is the loss of stabilization of processed *rps7* and *ndhB* transcripts in plastids, which leads to loss of the NDH complex (Hashimoto et al. 2003). Thus, it can be hypothesized that CRR2–RNA interactions for *matK*, *rpl23*, *rpoC2*, and *ycf2* are examples of neutral CNE interactions. Other examples include editing factors that are not essential for plant survival. The loss of the mitochondrial editing factor MEF12 and concomitant loss of editing at site *nad5*-374 does not lead to a detectable phenotype (Hartel et al. 2013a). Similarly, MEF10 mutants do not edit mitochondrial *nad2*-842 and fail to show phenotypic differences from the wild-type (Härtel et al. 2013b). In general, several editing sites are not conserved in evolution, flickering between C and T over time, which speaks against their functional importance and suggests that it does not matter whether a T is already encoded at the genome level or a U is established at the RNA level—arguing against editing being regulatory in these cases (Tillich et al. 2006b, 2006a, 2009b; Wu and Chaw 2022). Indeed, mutation of C → T edits at the genomic level is faster than that of nonedited Cs, which has been taken as evidence that editing confers little if any selective advantage at most sites (Shields and Wolfe 1997). Again, one of the most striking examples supporting the CNE theory is *psaA trans-*splicing in Chlamydomonas. Despite the involvement of many nucleus-encoded factors, the entire process was readily bypassed via the introduction of an intron-less *psaA* gene into the chloroplast genome (Lefebvre-Legendre et al. 2014). This uncoupled *psaA* expression from splicing factors and allowed wild-type growth under all tested growth conditions. Thus, *trans*-splicing per se does not fulfill an important regulatory function.

A prerequisite for CNE is that evolution provides a constant stream of available nuclear factors for resolving problems arising from organellar mutations. As mentioned above, helical repeat protein families have expanded across different taxa. For example, in the case of RF-like PPR proteins (discussed above in the section on CMS), specific genomic loci constantly spawn novel family members (Fujii et al. 2011), suggesting that novel editing factors arise from gene duplications (Wu and Chaw 2022). Such recombination events and duplications are considered to be the main reservoir for CNE processes to date.

### Evidence for regulation: start codon editing—regulation, translational regulation, and protein diversity

Despite the copious amounts of RNA maturation in plant organelles, and the many references to “regulation” of gene expression by RNA maturation factors in the literature, there is little published evidence that RNA maturation controls the rate of accumulation of organelle gene products under natural conditions. Evidence that RNA maturation factors are rate-limiting for gene expression is scarce, and evidence that the levels or activities of RNA maturation factors are modulated in response to changes in external conditions or feedback from organelle function is scarcer still. Thus, using a strict definition of regulation (Pichersky 2005), the degree to which RNA maturation controls organelle gene expression is very much an open question, despite the large number of potentially regulatory steps. The best evidence comes from studies on Chlamydomonas, where RNA maturation factors are implicated in “control by epistasy of synthesis” (CES), a regulatory mechanism that serves to avoid nonstoichiometric expression of subunits of the same complex (reviewed in Choquet and Wollman 2009). In the best-studied example, unassembled cytochrome *f* interacts with and induces the degradation of MCA1, an RNA stability factor for the *petA* mRNA encoding cytochrome *f* (Boulouis et al. 2011). This acts as a feedback loop that avoids the accumulation of excess unassembled cytochrome *f.* CES acts as a feedback control mechanism on expression of subunits for several complexes in Chlamydomonas chloroplasts (Wostrikoff et al. 2004; Drapier et al. 2007; Boulouis et al. 2011; Wietrzynski et al. 2021) and in yeast mitochondria (Herrmann et al. 2013), although the exact molecular details (and whether it is RNA maturation or translation initiation that is the control point) often remains unclear. To what extent CES operates in land plant chloroplasts is still unclear (Chotewutmontri et al. 2020; Wostrikoff and Stern 2007).

A different potential form of regulation of gene expression via RNA maturation is RNA editing, especially via the creation or removal of start and stop codons. In plants with a lot of start and stop codon editing it can be shown that these editing events are far more conserved than other editing events (Li et al. 2018), implying they confer an adaptive advantage. This may be because they provide a regulatory opportunity to control the timing and level of expression of the gene product, but may also simply be due to the opportunity this affords to avoid premature translation of partially edited mRNAs, as discussed above. Disentangling these 2 potential explanations will be difficult.

An alternative reason for the lack of studies demonstrating true regulation of plant organellar gene expression is that most such studies are conducted under laboratory conditions and only test single stressors. In natural settings, multiple environmental parameters are likely to change, and it is possible that organellar RNA processing is used to react to such changes. A rare in situ study of *Arabidopsis halleri* in a natural ecosystem demonstrated seasonal genome-wide differences in gene expression, including differential expression of plant organellar RNA processing factors (Nagano et al. 2019). Another example is the altered environmental adaptation of mutants of RNA processing factors, such as helicases and RBPs (Nawaz and Kang 2017; Lee and Kang 2020). Given the regulatory challenges of conducting field studies of genetically modified plants in Europe and other parts of the world, it will be some time before we have a more comprehensive understanding of how plant organellar RNA maturation is regulated under real-world conditions.

### RNA maturation and synthetic biology

Chloroplasts (and to a much lesser extent, mitochondria) make an excellent “chassis” for synthetic biology due to their small, well-understood genomes, and prodigious production potential (Boehm and Bock 2019; Bock 2022). Most chloroplast synbio projects have used natural control elements to drive expression of transgenes and thus are reliant on the host factors for correct expression of the desired products. In many cases, it would be preferable to use synthetic control elements that act orthogonally to the natural on- or off-switches of gene expression such that the intended products can be produced independently of them. Research to achieve this is in its infancy, but some exciting progress is being made. Although some effort is being put into developing transcriptional control of chloroplast transgenes (Piccinini et al. 2022), the majority of the effort so far mimics the endogenous chloroplast gene expression system by relying on RNA maturation to control gene expression. PPR proteins in particular lend themselves to rational design because each repeat unit binds a single nucleotide and the specificity-determining residues are known (Filipovska and Rackham 2013; Yagi et al. 2014). Synthetic PPR proteins with predictable binding affinities have been designed and tested by several groups with considerable success (Coquille et al. 2014; Gully et al. 2015; Shen et al. 2015, 2016; Yan et al. 2017; Miranda et al. 2018; Spåhr et al. 2018; Yu et al. 2019; Bernath-Levin et al. 2021; Manavski et al. 2021; Royan et al. 2021; Lesch et al. 2022). When expressed in chloroplasts, such proteins bind avidly to the intended target, and for example, can be used for RNA capture (McDermott et al. 2019). It is a small step from there to design proteins that can influence RNA maturation in desired ways.

### Promoting expression of target RNAs with synthetic helical repeat proteins

An inducible chloroplast expression system using a helical repeat protein was described for Chlamydomonas (Surzycki et al. 2007). The TPR protein NAC2, which is required for the stabilization of *psbD* mRNA (Boudreau et al. 2000; Ossenbühl and Nickelsen 2000), was used to regulate the expression of foreign genes under the control of the *psbD* 5′ UTR. For this purpose, the *NAC2* gene was placed under the control of the copper-repressible cytochrome *c6* promoter in the nuclear genome of Chlamydomonas strain *nac2-26* (Kuchka et al. 1989; Surzycki et al. 2007). Upon the depletion of copper ions, expression of the foreign gene driven by the *psbD* 5′ UTR was induced in an NAC2-dependent manner. In a related system, the cytochrome *c6* promoter was replaced by the vitamin-repressible *MetE* promoter and a thiamine pyrophosphate-responsive riboswitch to control NAC2 expression (Croft et al. 2007; Helliwell et al. 2014; Ramundo and Rochaix 2015). By supplying vitamin B_12_ and thiamine to the growth medium, the expression of NAC2 and proteins encoded by the transgenes under the control of the *psbD* 5′ UTR could be reversibly inactivated.

**Figure 5.**
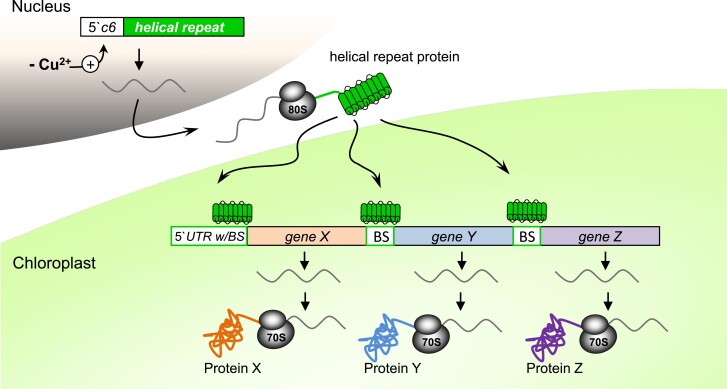
Helical repeat proteins for an optimized, synchronized, and regulated expression of transgenes in organelles. The expression of native or designer helical repeat proteins encoded in the nucleus under control of the *c6* promoter can be induced specifically by copper depletion. After its translation in the cytosol, the protein is imported into the organelle, where it interacts with BS to regulate the expression of a synthetic operon encoding the proteins X, Y, and Z.

Such inducible promoters in combination with binding sequences of native or artificial helical repeat proteins, as minimal intercistronic expression elements, may be used for the stabilization and translation of transgenes in synthetic operons (Fig. 5; Legen et al. 2018). This could be particularly helpful for the synchronous expression of functional units required for the balanced translation of introduced metabolic pathways. Moreover, the use of artificial PRR/OPR/TPR proteins designed to recognize certain RNA sequences that do not occur in the target genome could enable the specific regulation of foreign genes and reduce the risk of unwanted homologous recombination events.

A particularly well-studied PPR protein, RNA stabilizer, and translation factor, PPR10, was modified to predictably and specifically bind a range of RNA sequences that differed from their original targets (Barkan et al. 2012; Miranda et al. 2017). This approach was used to create a synthetic PPR10 that could bind a variant of the natural *atpH* 5′ UTR sequence (Rojas et al. 2019). When fused upstream of a GFP marker gene, this variant *atpH* 5′ UTR conferred synthetic-PPR10-dependent expression of GFP. As the synthetic PPR10 specifically recognized the variant *atpH* 5′ UTR and none of the endogenous mRNAs, and since the endogenous PPR10 could not bind to this sequence, the method provided an orthogonal “switch” that was largely independent of the host's regulation of chloroplast gene expression (Rojas et al. 2019). Indeed, this approach was used to drive GFP expression even in nonphotosynthetic tissues by expressing synthetic PPR10 from a suitable promoter (Yu et al. 2019). Other synthetic PPR proteins that can act as RNA stabilizers have also been created (Manavski et al. 2021).

Alternative approaches to promote the expression of a target RNA can be envisaged via other aspects of RNA maturation. For example, synthetic RNA editing factors can be designed to promote the activity of the desired gene product by altering the coding sequence, as demonstrated by complementation of an *rpoA*-editing mutant with a synthetic editing factor (Royan et al. 2021). Although this approach has not yet been used to activate the expression of chloroplast transgenes, the approach seems feasible. Indeed, it would seem possible to not only alter the coding sequence but also create start codons to switch on translation of the target RNA at will.

### Manipulating target RNAs with synthetic PPR proteins

For some applications, it is necessary to prevent expression of the target RNA rather than to promote it. This can also be achieved by use of synthetic RBPs, which represents an alternative to reverse genetic approaches that are difficult in plant organellar genomes. For example, the PPR protein RPF2 was modified to switch its target from the 5′ UTR of *cox3* to the coding sequence of *nad6*. Expression of this modified PPR in Arabidopsis mitochondria induced cleavage of *nad6* mRNA and a dramatic decrease in Nad6 protein and assembled Complex I (Colas des Francs-Small et al. 2018). This approach could be used to create synthetic *Rf* genes for use in controlling CMS in hybrid breeding systems, with the advantage that in theory the new *Rf*-gene variants could be created by single-nucleotide base editing of host RFL genes rather than requiring introduction of a transgene. Again, alternative ways of preventing expression of a target RNA can be envisaged, for example, introduction of a stop codon via RNA editing, or expression of a dominant negative factor that competes with a factor required for RNA stabilization or initiation of translation.

### Outlook

Much progress has been made in understanding the complex RNA maturation systems in plant organelles, but much remains to be discovered. The considerable (and necessary) efforts that have been put into cataloging the numerous RBPs involved need to be complemented by equivalent efforts at understanding the processes mechanistically. There are several particular areas that we would encourage researchers to focus on. Our understanding of the kinetics of RNA maturation in organelles is still rudimentary. A much better understanding of the rates of the different processes (and the rate-limiting steps within them) are needed to discover which have regulatory potential. New labeling techniques that can provide high-throughput pulse-chase analysis appear to offer a way forward (Szabo et al. 2020). With respect to the macromolecular interactions involved, nearly all the focus has been on the RBPs and their interaction with RNA, but little attention has been paid to RNA–RNA interactions (either inter- or intramolecular) which are surely also extremely important. Again, new technologies for experimentally probing RNA structures exist (Strobel et al. 2018), but application to plant organellar RNAs is just starting (Gawroński et al. 2020). Building on this last point, the larger-scale organization of the RNA maturation machinery within organelle nucleoids is a very open question. There is a lot of active research in other genetic systems looking at the structure and activities of ribonucleoprotein “granules” using new approaches that could be highly relevant to plant organelles (Wiedner and Giudice 2021). Pulling together the organellar variety of RBPs and RNAs into granular structures could facilitate RNA maturation analogous to what was already observed in human mitochondria (Jourdain et al. 2016). In terms of genomic engineering a plastid genome as an expression hub for high-value proteins, removing the necessity of any RNA processing, e.g. starting by removing all introns and editing sites, could be an exciting research avenue. Finally, new avenues into finding regulatory roles for RNA maturation in organelles may be found in tissues beyond leaves—a case in point is the putative role of mitochondrial gene expression in regulating seed germination and early seedling establishment (Best et al. 2020). Thus several opportunities for exciting breakthroughs are on the horizon.

## Supplemental data

The following materials are available in the online version of this article.

**Supplemental Table S1**. Gene and RBP numbers of selected species, supports Fig. 1.

**Supplemental Table S2**. PPR proteins involved in plant organellar RNA splicing, supports Chapter 4.1

## Supplementary Material

koad049_Supplementary_Data

## Data Availability

No data were generated in the preparation of this review.
