# Supplementary material for: Plant organellar RNA maturation
Source: Plant Cell. 2023 Feb 21;35(6):1727–51. doi: 10.1093/plcell/koad049 (PMC10226603; doi:10.1093/plcell/koad049)
Supplement: koad049_Supplementary_Data [file koad049_supplementary_data.pdf]

Table S1: Gene and RBP numbers of selected species. Supports Fig. 1.

Species	Gene number	Number of RBPs	RBPs/ gene
S. cerevisiae **(nucleus)**	**6275** §	**1273** (Hentze et al., 2018)	0.20
S. cerevisiae **(mitochondria)**	**35** (Malina et al., 2018)	**210** ¥	6.00
C. elegans **(nucleus)**	**20512** (Spieth et al., 2014)	**593** (Hentze et al., 2018)	0.03
Drosophila **(nucleus)**	**13601** (Adams et al., 2000)	**777** (Hentze et al., 2018)	0.06
M. musculus **(nucleus)**	**46206** (Bult et al., 2016)	**1914** (Hentze et al., 2018)	0.04
M. musculus **(mitochondria)**	**73** (Bibb et al., 1981)	**71** #	0.97
H. sapiens **(nucleus)**	**46880** $	**1393** (Hentze et al., 2018)	0.04
H. sapiens **(mitochondria)**	**73** (Anderson et al., 1981)	**71** #	0.97
A. ***thaliana*** **(nucleus)**	**25500** (Project, 2000)	**719** (Hentze et al., 2018)	0.03
A. ***thaliana*** **(chloroplast)**	**117** (Ahmad et al., 2015)	**179**&	1.53
A. ***thaliana*** **(mitochondria)**	**57** (Unseld et al., 1997)	**376**&	6.60
C. reinhardtii (chloroplast)	**120** (Maul et al., 2002)	**138**£	1.15

§ saccharomyces Genome database; https://www.yeastgenome.org/

¥ according to EMBL RBPbase; https://rbpbase.shiny.embl.de/

mitocharta 3.0; GO category “RNA metabolism” (Rath et al., 2021)

$ Statistics about the current GENCODE Release (version 37) of the human genome. Available online: https://www.gencodegenes.org/human/stats.html (accessed on 22 July 2022).

& see Table 1

£ In *Chlamydomonas*, no comparable analysis to detect the total number of RBPs has been conducted. However, only based on the most prominent families of RNA binding proteins in the alga, i.e. PPRs, OPRs and TPR-HAT proteins, the ratio for RBS/gene is roughly estimated to be > 1.15 for the chloroplast (Tourasse et al., 2013; Bohne et al., 2016; Cerutti, Bohne, Rochaix, Vallon, unpublished data).

References for Table S1

Adams, M.D., Celniker, S.E., Holt, R.A., Evans, C.A., Gocayne, J.D., Amanatides, P.G., Scherer, S.E., Li, P.W., Hoskins, R.A., Galle, R.F., George, R.A., Lewis, S.E., Richards, S., Ashburner, M., Henderson, S.N., Sutton, G.G., Wortman, J.R., Yandell, M.D., Zhang, Q., Chen, L.X., Brandon, R.C., Rogers, Y.H., Blazej, R.G., Champe, M., Pfeiffer, B.D., Wan, K.H., Doyle, C., Baxter, E.G., Helt, G., Nelson, C.R., Gabor, G.L., Abril, J.F., Agbayani, A., An, H.J., Andrews-

Pfannkoch, C., Baldwin, D., Ballew, R.M., Basu, A., Baxendale, J., Bayraktaroglu, L., Beasley, E.M., Beeson, K.Y., Benos, P.V., Berman, B.P., Bhandari, D., Bolshakov, S., Borkova, D., Botchan, M.R., Bouck, J., Brokstein, P., Brottier, P., Burtis, K.C., Busam, D.A., Butler, H., Cadieu, E., Center, A., Chandra, I., Cherry, J.M., Cawley, S., Dahlke, C., Davenport, L.B., Davies, P., de Pablos, B., Delcher, A., Deng, Z., Mays, A.D., Dew, I., Dietz, S.M., Dodson, K., Doup, L.E., Downes, M., Dugan-Rocha, S., Dunkov, B.C., Dunn, P., Durbin, K.J., Evangelista, C.C., Ferraz, C., Ferriera, S., Fleischmann, W., Fosler, C., Gabrielian, A.E., Garg, N.S., Gelbart, W.M., Glasser, K., Glodek, A., Gong, F., Gorrell, J.H., Gu, Z., Guan, P., Harris, M., Harris, N.L., Harvey, D., Heiman, T.J., Hernandez, J.R., Houck, J., Hostin, D., Houston, K.A., Howland, T.J., Wei, M.H., Ibegwam, C., Jalali, M., Kalush, F., Karpen, G.H., Ke, Z., Kennison, J.A., Ketchum, K.A., Kimmel, B.E., Kodira, C.D., Kraft, C., Kravitz, S., Kulp, D., Lai, Z., Lasko, P., Lei, Y., Levitsky, A.A., Li, J., Li, Z., Liang, Y., Lin, X., Liu, X., Mattei, B., McIntosh, T.C., McLeod, M.P., McPherson, D., Merkulov, G., Milshina, N.V., Mobarry, C., Morris, J., Moshrefi, A., Mount, S.M., Moy, M., Murphy, B., Murphy, L., Muzny, D.M., Nelson, D.L., Nelson, D.R., Nelson, K.A., Nixon, K., Nusskern, D.R., Pacleb, J.M., Palazzolo, M., Pittman, G.S., Pan, S., Pollard, J., Puri, V., Reese, M.G., Reinert, K., Remington, K., Saunders, R.D., Scheeler, F., Shen, H., Shue, B.C., Siden-Kiamos, I., Simpson, M., Skupski, M.P., Smith, T., Spier, E., Spradling, A.C., Stapleton, M., Strong, R., Sun, E., Svirskas, R., Tector, C., Turner, R., Venter, E., Wang, A.H., Wang, X., Wang, Z.Y., Wassarman, D.A., Weinstock, G.M., Weissenbach, J., Williams, S.M., WoodageT, Worley, K.C., Wu, D., Yang, S., Yao, Q.A., Ye, J., Yeh, R.F., Zaveri, J.S., Zhan, M., Zhang, G., Zhao, Q., Zheng, L., Zheng, X.H., Zhong, F.N., Zhong, W., Zhou, X., Zhu, S., Zhu, X., Smith, H.O., Gibbs, R.A., Myers, E.W., Rubin, G.M., and Venter, J.C. (2000). The genome sequence of Drosophila melanogaster. Science **287,** 2185-2195.

Ahmad, N.A., Zeshan, M., Gill, S.S., and Mehmood, M.A. (2015). Functional Genomic Studies of Chloroplast Genome in Arabidopsis. In Arabidopsis thaliana: Cultivation, Life Cycle and Functional Genomics, A. Sofo, ed (USA: Nova Science Publishers.

Anderson, S., Bankier, A.T., Barrell, B.G., de Bruijn, M.H., Coulson, A.R., Drouin, J., Eperon, I.C., Nierlich, D.P., Roe, B.A., Sanger, F., Schreier, P.H., Smith, A.J., Staden, R., and Young, I.G. (1981). Sequence and organization of the human mitochondrial genome. Nature **290,** 457-465.

Bibb, M.J., Van Etten, R.A., Wright, C.T., Walberg, M.W., and Clayton, D.A. (1981). Sequence and gene organization of mouse mitochondrial DNA. Cell **26,** 167-180.

Bohne, A.V., Schwenkert, S., Grimm, B., and Nickelsen, J. (2016). Roles of Tetratricopeptide Repeat Proteins in Biogenesis of the Photosynthetic Apparatus. Int Rev Cell Mol Biol **324,** 187-227.

Bult, C.J., Eppig, J.T., Blake, J.A., Kadin, J.A., Richardson, J.E., and Mouse Genome Database, G. (2016). Mouse genome database 2016. Nucleic Acids Res **44,** D840-847.

Hentze, M.W., Castello, A., Schwarzl, T., and Preiss, T. (2018). A brave new world of RNA-binding proteins. Nat Rev Mol Cell Biol **19,** 327-341.

Malina, C., Larsson, C., and Nielsen, J. (2018). Yeast mitochondria: an overview of mitochondrial biology and the potential of mitochondrial systems biology. FEMS Yeast Res **18**.

Maul, J.E., Lilly, J.W., Cui, L., dePamphilis, C.W., Miller, W., Harris, E.H., and Stern, D.B. (2002). The *Chlamydomonas reinhardtii* plastid chromosome: islands of genes in a sea of repeats. Plant Cell **14,** 2659-2679.

Project, T.A.G. (2000). Analysis of the genome sequence of the flowering plant *Arabidopsis thaliana.* Nature **408,** 796-815.

Rath, S., Sharma, R., Gupta, R., Ast, T., Chan, C., Durham, T.J., Goodman, R.P., Grabarek, Z., Haas, M.E., Hung, W.H.W., Joshi, P.R., Jourdain, A.A., Kim, S.H., Kotrys, A.V., Lam, S.S., McCoy, J.G., Meisel, J.D., Miranda, M., Panda, A., Patgiri, A., Rogers, R., Sadre, S., Shah, H., Skinner, O.S., To, T.L., Walker, M.A., Wang, H., Ward, P.S., Wengrod, J., Yuan, C.C., Calvo,

S.E., and Mootha, V.K. (2021). MitoCarta3.0: an updated mitochondrial proteome now with sub-organelle localization and pathway annotations. Nucleic Acids Res **49,** D1541-D1547.
Spieth, J., Lawson, D., Davis, P., Williams, G., and Howe, K. (2014). Overview of gene structure in C. elegans (29. Oktober 2014). In WormBook, T.C.e.R. Community, ed.
Tourasse, N.J., Choquet, Y., and Vallon, O. (2013). PPR proteins of green algae. RNA Biol **10,** 1526-1542.
Unseld, M., Marenfeld, J., Brandt, P., and Brennicke, A. (1997). The mitochondrial genome of *Arabidopsis thaliana* contains 57 genes in 366,924 nucleotides. Nat. Genet. **15,** 57-61.

Table S2: PPR proteins involved in plant organellar RNA splicing. Supports chapter 4.1.

PPR Name[§]	Intron[$]	Reference
**AtABO5**	*nad2i3*	(Liu et al., 2010)
**AtBIR6**	*nad7i1*	(Koprivova et al., 2010)
**ZmDEK2**	*nad1i1*	(Qi et al., 2017)
**ZmDEK35**	*nad4i1*	(Chen et al., 2017)
**ZmDEK37**	*nad2i1*	(Dai et al., 2018)
**ZmDEK41 / ZmDEK43/OsPPR5**	*nad4i3*	(Zhu et al., 2019) / (Ren et al., 2020) / (Zhang et al., 2021a)
**ZmEMP8**	*nad1i4, nad4i1*	(Sun et al., 2018)
**ZmEMP11**	*nad1*	(Ren et al., 2017)
**ZmEMP12**	*nad2*	(Sun et al., 2019)
**ZmEMP16**	*nad2i4*	(Xiu et al., 2016)
**ZmEMP32**	*nad7i2*	(Yang et al., 2021)
**ZmEMP602 / ZmEMP24**	*nad4i1,3*	(Ren et al., 2019) / (Xiu et al., 2020)
**ZmEMP25**	*nad5i1,2,3*	(Xiu et al., 2020)
**ZmEMP603**	*nad1i2*	(Fan et al., 2021)
**OsFLO10**	*nad1i1*	(Wu et al., 2019)
**AtMID1**	*nad2i1*	(Zhao et al., 2020)
**AtMISF2 / ZmEMP10**	*nad2i1*	(Nguyen et al., 2022) / (Cai et al., 2017)
**AtMISF26**	*nad2i3*	(Wang et al., 2018a)
**AtMISF68**	*nad2i2, nad4i1*	(Wang et al., 2018a)
**AtMISF74**	*nad1i4, nad2i4*	(Wang et al., 2018a)

**AtMTL1**	*nad7i2*	(Haïli et al., 2016)
**OsNBL3**	*nad5i4*	(Qiu et al., 2021)
**AtOTP43**	*nad1i1*	(Falcon de Longevialle et al., 2007)
**AtOTP439**	*nad5i2*	(Colas des Francs-Small et al., 2014)
**ZmPPR101**	*nad5i1, nad5i2*	(Yang et al., 2020a)
**ZmPPR14**	*nad2i3, nad7i1, nad7i2*	(Wang et al., 2020a)
**ZmPPR18**	*nad4i1*	(Liu et al., 2020)
**ZmPPR20**	*nad2i3*	(Yang et al., 2020b)
**PpPPR_43**	*cox1i3*	(Ichinose et al., 2012)
**ZmPPR231**	*nad2i3, nad5*	(Yang et al., 2020a)
**ZmPPR278**	*nad2i4, nad5i1i4*	(Yang et al., 2022a)
**ZmPPR-SMR1**	*many*	(Cao et al., 2022)
**OsRL1**	*nad4i1*	(Wu et al., 2020)
**AtRTP7**	*nad7i1*	(Yang et al., 2022b)
**AtSLO3**	*nad7i2*	(Hsieh et al., 2015)
**AtSLO4**	*nad2i1*	(Weißenberger et al., 2017)
**ZmSMK9**	*nad5*	(Pan et al., 2019)
**ZmSPR2**	*many*	(Cao et al., 2022)
**AtTANG2/ZmEMP25/OsPPR939**	*nad5*	(Colas des Francs-Small et al., 2014) / (Xiu et al., 2020) / (Zheng et al., 2021)
**OsCDE4**	***rpl2, ndhA, ndhB***	(Liu et al., 2021)
**AtECD2**	*ndhA, rps12-2, clpP-2, ycf3-1*	(Wang et al., 2021)
**AtEMB1270**	***ndhA, ndhB, clpP-2, ycf3-1***	(Zhang et al., 2021b)
**AtEMB2654 / ZmEMB-7L**	***rps12**-1 / atpF, rpl2, ndhA, ndhB, rps12-1, ycf3-2*	(Lee et al., 2019; Aryamanesh et al., 2017) / (Yuan et al., 2019)
**AtOTP51 / OsOTP51**	*atpF, ycf3-2 / atpF, petB, rps16, ndhA, ycf3-2*	(de Longevialle et al., 2008) / (Ye et al., 2012)
**AtOTP70**	*rpoC1*	(Chateigner-Boutin et al., 2011)
**AtPBF2**	***ycf3-1***	(Wang et al., 2020b)
**AtPDM1**	***ndhA**, **trnK***	(Zhang et al., 2015)
**AtPDM3**	*ndhB, trnA, clpP-1*	(Zhang et al., 2017)
**AtPDM4**	***petB**, **petD**, **ndhA**, clpP-1, **ycf3-1***	(Wang et al., 2020c)
**OsPGL12**	*ndhA*	(Chen et al., 2019)

**ZmPPR4 / AtPPR4 / OsPPR4**	***rps12-2*** **/** *ndhA, ndhB,* ***rps12-1****, rps12-2* **/** *rps12-1, rps12-2, ndhA, atpF, petB*	(Schmitz-Linneweber et al., 2006) / (Tadini et al., 2018; Lee et al., 2019) / (Lee et al., 2019)
**ZmPPR5**	***trnG***	(Beick et al., 2008)
**OsPPR6**	*ycf3-1*	(Tang et al., 2017)
**PpPPR_38**	*clpP*	(Hattori et al., 2007)
**PpPPR66 / AtPPR66**	*ndhA / ndhA*	(Ito et al., 2018) / (Ito et al., 2018)
**ZmPPR467**	*rpl2, atpF, ndhA, ndhB, ycf3-2*	(Zhao et al., 2021)
**OsSLA4**	*atpF, rpl2, petB, rpl16, ndhA, trnG, rps12-2*	(Wang et al., 2018b)
**OsSLC1**	*rps16*	(Lv et al., 2020)
**AtSOT5**	*rpl2, trnK*	(Huang et al., 2018)
**OsWSL**	*rpl2*	(Tan et al., 2014)
**OsWSL4**	*atpF, rpl2, ndaH, rps12-2*	(Wang et al., 2017)
**OsWSL5**	*rpl2, rps12-1*	(Liu et al., 2018)
**ZmTha8 / AtTHA8**	***trnA, ycf3-1*** **/** *trnA, ycf3-1*	(Khrouchtchova et al., 2012)

§ grey shading = mitochondrial PPR protein splicing factor; green shading = chloroplast PPR protein splicing factor

$ green chloroplast gene names = experimental support for PPR-RNA interaction

References for Suppl. Tab. 2

Aryamanesh, N., Ruwe, H., Sanglard, L.V., Eshraghi, L., Bussell, J.D., Howell, K.A., Small, I., and des Francs-Small, C.C. (2017). The Pentatricopeptide Repeat Protein EMB2654 Is Essential for Trans-Splicing of a Chloroplast Small Ribosomal Subunit Transcript. Plant Physiol. **173**: 1164–1176.

Beick, S., Schmitz-Linneweber, C., Williams-Carrier, R., Jensen, B., and Barkan, A. (2008). The pentatricopeptide repeat protein PPR5 stabilizes a specific tRNA precursor in maize chloroplasts. Mol. Cell. Biol. **28**: 5337–5347.

Cai, M., Li, S., Sun, F., Sun, Q., Zhao, H., Ren, X., Zhao, Y., Tan, B.-C., Zhang, Z., and Qiu, F. (2017). *Emp10*encodes a mitochondrial PPR protein that affects the*cis*-splicing of*nad2*intron 1 and seed development in maize. Plant J. **91**: 132–144.

Cao, S.-K., Liu, R., Wang, M., Sun, F., Sayyed, A., Shi, H., Wang, X., and Tan, B.-C. (2022). The small PPR protein SPR2 interacts with PPR-SMR1 to facilitate the splicing of introns in maize mitochondria. Plant Physiol.

Chateigner-Boutin, A.-L., Colas des Francs-Small, C., Delannoy, E., Kahlau, S., Tanz, S.K., Falcon de Longevialle, A., Fujii, S., and Small, I. (2011). OTP70 is a pentatricopeptide repeat protein of the E subgroup involved in splicing of the plastid transcript *rpoC1*. Plant J. **65**: 532–542.

Chen, L. et al. (2019). PALE-GREEN LEAF12 Encodes a Novel Pentatricopeptide Repeat Protein Required for Chloroplast Development and 16S rRNA Processing in Rice. Plant Cell Physiol. **60**: 587–598.

Chen, X., Feng, F., Qi, W., Xu, L., Yao, D., Wang, Q., and Song, R. (2017). Dek35 Encodes a PPR Protein that Affects cis -Splicing of Mitochondrial nad4 Intron 1 and Seed Development in Maize. Molecular Plant **10**: 427–441.

Colas des Francs-Small, C., Falcon de Longevialle, A., Li, Y., Lowe, E., Tanz, S.K., Smith, C., Bevan, M.W., and Small, I. (2014). The pentatricopeptide repeat proteins TANG2 and ORGANELLE TRANSCRIPT PROCESSING439 are involved in the splicing of the multipartite *nad5* transcript encoding a subunit of mitochondrial complex I. Plant Physiol. **165**: 1409–1416.

Dai, D., Luan, S., Chen, X., Wang, Q., Feng, Y., Zhu, C., Qi, W., and Song, R. (2018). Maize Dek37 Encodes a P-type PPR Protein That Affects cis-Splicing of Mitochondrial nad2 Intron 1 and Seed Development. Genetics **208**: 1069–1082.

Falcon de Longevialle, A., Meyer, E.H., Andrés, C., Taylor, N.L., Lurin, C., Millar, A.H., and Small, I.D.

(2007). The pentatricopeptide repeat gene OTP43 is required for trans-splicing of the mitochondrial *nad1* intron 1 in *Arabidopsis thaliana*. Plant Cell **19**: 3256–3265.

Fan, K., Ren, Z., Zhang, X., Liu, Y., Fu, J., Qi, C., Tatar, W., Rasmusson, A.G., Wang, G., and Liu, Y. (2021). The pentatricopeptide repeat protein EMP603 is required for the splicing of mitochondrial Nad1 intron 2 and seed development in maize. J. Exp. Bot. **72**: 6933–6948.

Haïli, N., Planchard, N., Arnal, N., Quadrado, M., Vrielynck, N., Dahan, J., des Francs-Small, C.C., and Mireau, H. (2016). The MTL1 Pentatricopeptide Repeat Protein Is Required for Both Translation and Splicing of the Mitochondrial NADH DEHYDROGENASE SUBUNIT7 mRNA in Arabidopsis. Plant Physiol. **170**: 354–366.

Hattori, M., Miyake, H., and Sugita, M. (2007). A Pentatricopeptide repeat protein is required for RNA processing of clpP Pre-mRNA in moss chloroplasts. J. Biol. Chem. **282**: 10773–10782.

Hsieh, W.-Y., Liao, J.-C., Chang, C.-Y., Harrison, T., Boucher, C., and Hsieh, M.-H. (2015). The SLOW GROWTH3 Pentatricopeptide Repeat Protein Is Required for the Splicing of Mitochondrial NADH Dehydrogenase Subunit7 Intron 2 in Arabidopsis. Plant Physiol. **168**: 490–501.

Huang, W., Zhu, Y., Wu, W., Li, X., Zhang, D., Yin, P., and Huang, J. (2018). The Pentatricopeptide Repeat Protein SOT5/EMB2279 Is Required for Plastid rpl2 and trnK Intron Splicing. Plant Physiol. **177**: 684–697.

Ichinose, M., Tasaki, E., Sugita, C., and Sugita, M. (2012). A PPR-DYW protein is required for splicing of a group II intron of cox1 pre-mRNA in Physcomitrella patens. Plant J. **70**: 271–278.

Ito, A., Sugita, C., Ichinose, M., Kato, Y., Yamamoto, H., Shikanai, T., and Sugita, M. (2018). An evolutionarily conserved P-subfamily pentatricopeptide repeat protein is required to splice the plastid ndhA transcript in the moss Physcomitrella patens and Arabidopsis thaliana. Plant J. **94**: 638–648.

Khrouchtchova, A., Monde, R.A., and Barkan, A. (2012). A short PPR protein required for the splicing of specific group II introns in angiosperm chloroplasts. RNA **18**: 1197–1209.

Koprivova, A., Colas des Francs-Small, C., Calder, G., Mugford, S.T., Tanz, S., Lee, B.-R., Zechmann, B., Small, I., and Kopriva, S. (2010). Identification of a pentatricopeptide repeat protein implicated in splicing of intron 1 of mitochondrial *nad7* transcripts. J. Biol. Chem. **285**: 32192–32199.

Lee, K., Park, S.J., Colas des Francs-Small, C., Whitby, M., Small, I., and Kang, H. (2019). The coordinated action of PPR4 and EMB2654 on each intron half mediates trans-splicing of rps12 transcripts in plant chloroplasts. Plant J. **100**: 1193–1207.

Liu, R., Cao, S.-K., Sayyed, A., Xu, C., Sun, F., Wang, X., and Tan, B.-C. (2020). The Mitochondrial Pentatricopeptide Repeat Protein PPR18 Is Required for the cis-Splicing of nad4 Intron 1 and Essential to Seed Development in Maize. Int. J. Mol. Sci. **21**.

Liu, X., Lan, J., Huang, Y., Cao, P., Zhou, C., Ren, Y., He, N., Liu, S., Tian, Y., Nguyen, T., Jiang, L., and Wan, J. (2018). WSL5, a pentatricopeptide repeat protein, is essential for chloroplast biogenesis in rice under cold stress. J. Exp. Bot. **69**: 3949–3961.

Liu, X., Zhang, X., Cao, R., Jiao, G., Hu, S., Shao, G., Sheng, Z., Xie, L., Tang, S., Wei, X., and Hu, P. (2021). CDE4 encodes a pentatricopeptide repeat protein involved in chloroplast RNA splicing and affects chloroplast development under low-temperature conditions in rice. J. Integr. Plant Biol. **63**: 1724–1739.

Liu, Y., He, J., Chen, Z., Ren, X., Hong, X., and Gong, Z. (2010). ABA overly-sensitive 5 (ABO5), encoding a pentatricopeptide repeat protein required for cis-splicing of mitochondrial nad2 intron 3, is involved in the abscisic acid response in Arabidopsis. The Plant Journal **63**: 749–765.

de Longevialle, A.F., Hendrickson, L., Taylor, N.L., Delannoy, E., Lurin, C., Badger, M., Millar, A.H., and Small, I. (2008). The pentatricopeptide repeat geneOTP51with two LAGLIDADG motifs is required for thecis-splicing of plastidycf3intron 2 inArabidopsis thaliana. Plant J. **56**: 157–168.

Lv, J. et al. (2020). OsSLC1 Encodes a Pentatricopeptide Repeat Protein Essential for Early Chloroplast Development and Seedling Survival. Rice **13**: 25.

Nguyen, T.-T., Best, C., Shevtsov, S., Zmudjak, M., Quadrado, M., Mizrahi, R., Zer, H., Mireau, H., and Ostersetzer-Biran, O. (2022). MISF2 Encodes an Essential Mitochondrial Splicing Cofactor Required for nad2 mRNA Processing and Embryo Development in Arabidopsis thaliana. Int. J. Mol. Sci. **23**.

Pan, Z., Liu, M., Xiao, Z., Ren, X., Zhao, H., Gong, D., Liang, K., Tan, Z., Shao, Y., and Qiu, F. (2019). ZmSMK9, a pentatricopeptide repeat protein, is involved in the cis-splicing of nad5, kernel development and plant architecture in maize. Plant Sci. **288**: 110205.

Qiu, T., Zhao, X., Feng, H., Qi, L., Yang, J., Peng, Y.-L., and Zhao, W. (2021). OsNBL3, a mitochondrion-localized pentatricopeptide repeat protein, is involved in splicing nad5 intron 4 and its disruption causes lesion mimic phenotype with enhanced resistance to biotic and abiotic stresses. Plant Biotechnol. J. **19**: 2277–2290.

Qi, W., Yang, Y., Feng, X., Zhang, M., and Song, R. (2017). Mitochondrial Function and Maize Kernel Development Requires Dek2, a Pentatricopeptide Repeat Protein Involved in nad1 mRNA Splicing. Genetics **205**: 239–249.

Ren, R.C., Wang, L.L., Zhang, L., Zhao, Y.J., Wu, J.W., Wei, Y.M., Zhang, X.S., and Zhao, X.Y. (2020). DEK43 is a P-type pentatricopeptide repeat (PPR) protein responsible for the Cis-splicing of nad4 in maize mitochondria. J. Integr. Plant Biol. **62**: 299–313.

Ren, X., Pan, Z., Zhao, H., Zhao, J., Cai, M., Li, J., Zhang, Z., and Qiu, F. (2017). EMPTY PERICARP11 serves as a factor for splicing of mitochondrial nad1 intron and is required to ensure proper seed development in maize. J. Exp. Bot. **68**: 4571–4581.

Ren, Z., Fan, K., Fang, T., Zhang, J., Yang, L., Wang, J., Wang, G., and Liu, Y. (2019). Maize Empty Pericarp602 Encodes a P-Type PPR Protein That Is Essential for Seed Development. Plant Cell Physiol. **60**: 1734–1746.

Schmitz-Linneweber, C., Williams-Carrier, R.E., Williams-Voelker, P.M., Kroeger, T.S., Vichas, A., and Barkan, A. (2006). A pentatricopeptide repeat protein facilitates the trans-splicing of the maize chloroplast rps12 pre-mRNA. Plant Cell **18**: 2650–2663.
Sun, F., Xiu, Z., Jiang, R., Liu, Y., Zhang, X., Yang, Y.-Z., Li, X., Zhang, X., Wang, Y., and Tan, B.-C. (2019). The mitochondrial pentatricopeptide repeat protein EMP12 is involved in the splicing of three nad2 introns and seed development in maize. J. Exp. Bot. **70**: 963–972.
Sun, F., Zhang, X., Shen, Y., Wang, H., Liu, R., Wang, X., Gao, D., Yang, Y.-Z., Liu, Y., and Tan, B.-C. (2018). The pentatricopeptide repeat protein EMPTY PERICARP8 is required for the splicing of three mitochondrial introns and seed development in maize. Plant J.
Tadini, L., Ferrari, R., Lehniger, M.K., Mizzotti, C., Moratti, F., Resentini, F., Colombo, M., Costa, A., Masiero, S., and Pesaresi, P. (2018). Trans-splicing of plastid rps12 transcripts, mediated by AtPPR4, is essential for embryo patterning in Arabidopsis thaliana. Planta **248**: 257–265.
Tang, J. et al. (2017). OsPPR6, a pentatricopeptide repeat protein involved in editing and splicing chloroplast RNA, is required for chloroplast biogenesis in rice. Plant Mol. Biol. **95**: 345–357.
Tan, J. et al. (2014). A novel chloroplast-localized pentatricopeptide repeat protein involved in splicing affects chloroplast development and abiotic stress response in rice. Mol. Plant **7**: 1329–1349.
Wang, C., Aubé, F., Quadrado, M., Dargel-Graffin, C., and Mireau, H. (2018a). Three new pentatricopeptide repeat proteins facilitate the splicing of mitochondrial transcripts and complex I biogenesis in Arabidopsis. J. Exp. Bot. **69**: 5131–5140.
Wang, H.-C., Chen, Z., Yang, Y.-Z., Sun, F., Ding, S., Li, X.-L., Xu, C., and Tan, B.-C. (2020a). PPR14 Interacts With PPR-SMR1 and CRM Protein Zm-mCSF1 to Facilitate Mitochondrial Intron Splicing in Maize. Front. Plant Sci. **11**: 814.
Wang, X., An, Y., Qi, Z., and Xiao, J. (2021). PPR protein Early Chloroplast Development 2 is essential for chloroplast development at the early stage of Arabidopsis development. Plant Sci. **308**: 110908.
Wang, X., Yang, Z., Zhang, Y., Zhou, W., Zhang, A., and Lu, C. (2020b). Pentatricopeptide repeat protein PHOTOSYSTEM I BIOGENESIS FACTOR2 is required for splicing of ycf3. J. Integr. Plant Biol. **62**: 1741–1761.
Wang, X., Zhao, L., Man, Y., Li, X., Wang, L., and Xiao, J. (2020c). PDM4, a Pentatricopeptide Repeat Protein, Affects Chloroplast Gene Expression and Chloroplast Development in Arabidopsis thaliana. Front. Plant Sci. **11**: 1198.
Wang, Y. et al. (2017). WHITE STRIPE LEAF4 Encodes a Novel P-Type PPR Protein Required for Chloroplast Biogenesis during Early Leaf Development. Front. Plant Sci. **8**: 1116.
Wang, Z.-W., Lv, J., Xie, S.-Z., Zhang, Y., Qiu, Z.-N., Chen, P., Cui, Y.-T., Niu, Y.-F., Hu, S.-K., Jiang, H.-Z., and Others (2018b). OsSLA4 encodes a pentatricopeptide repeat protein essential for early chloroplast development and seedling growth in rice. Plant Growth Regul. **84**: 249–260.
Weißenberger, S., Soll, J., and Carrie, C. (2017). The PPR protein SLOW GROWTH 4 is involved in editing of nad4 and affects the splicing of nad2 intron 1. Plant Mol. Biol. **93**: 355–368.
Wu, M. et al. (2019). Rice FLOURY ENDOSPERM10 encodes a pentatricopeptide repeat protein that is essential for the trans-splicing of mitochondrial nad1 intron 1 and endosperm development. New Phytol. **223**: 736–750.
Wu, M.-W., Zhao, H., Zhang, J.-D., Guo, L., and Liu, C.-M. (2020). RADICLELESS 1 (RL1)-mediated nad4 intron 1 splicing is crucial for embryo and endosperm development in rice (Oryza sativa L.). Biochem. Biophys. Res. Commun. **523**: 220–225.
Xiu, Z., Peng, L., Wang, Y., Yang, H., Sun, F., Wang, X., Cao, S.-K., Jiang, R., Wang, L., Chen, B.-Y., and Tan, B.-C. (2020). Empty Pericarp24 and Empty Pericarp25 Are Required for the Splicing of Mitochondrial Introns, Complex I Assembly, and Seed Development in Maize. Front. Plant Sci. **11**.
Xiu, Z., Sun, F., Shen, Y., Zhang, X., Jiang, R., Bonnard, G., Zhang, J., and Tan, B.-C. (2016). EMPTY PERICARP16 is required for mitochondrial nad2 intron 4 cis-splicing, complex I assembly and seed development in maize. Plant J. **85**: 507–519.
Yang, H., Xiu, Z., Wang, L., Cao, S.-K., Li, X., Sun, F., and Tan, B.-C. (2020a). Two Pentatricopeptide Repeat Proteins Are Required for the Splicing of nad5 Introns in Maize. Front. Plant Sci. **11**: 732.
Yang, J., Cui, Y., Zhang, X., Yang, Z., Lai, J., Song, W., Liang, J., and Li, X. (2022a). Maize PPR278 Functions in Mitochondrial RNA Splicing and Editing. Int. J. Mol. Sci. **23**.
Yang, Y.-Z., Ding, S., Liu, X.-Y., Tang, J.-J., Wang, Y., Sun, F., Xu, C., and Tan, B.-C. (2021). EMP32 is required for the cis-splicing of nad7 intron 2 and seed development in maize. RNA Biol. **18**: 499–509.
Yang, Y.-Z., Ding, S., Wang, Y., Wang, H.-C., Liu, X.-Y., Sun, F., Xu, C., Liu, B., and Tan, B.-C. (2020b). PPR20 Is Required for the cis-Splicing of Mitochondrial nad2 Intron 3 and Seed Development in Maize. Plant Cell Physiol. **61**: 370–380.
Yang, Y., Zhao, Y., Zhang, Y., Niu, L., Li, W., Lu, W., Li, J., Schäfer, P., Meng, Y., and Shan, W. (2022b). A mitochondrial RNA processing protein mediates plant immunity to a broad spectrum of pathogens by modulating the mitochondrial oxidative burst. Plant Cell **34**: 2343–2363.
Ye, J.-W., Gong, Z.-Y., Chen, C.-G., Mi, H.-L., and Chen, G.-Y. (2012). A mutation of OSOTP 51 leads to impairment of photosystem I complex assembly and serious photo-damage in rice. J. Integr. Plant Biol. **54**: 87–98.
Yuan, N., Wang, J., Zhou, Y., An, D., Xiao, Q., Wang, W., and Wu, Y. (2019). EMB-7L is required for embryogenesis and plant development in maize involved in RNA splicing of multiple chloroplast genes. Plant Sci. **287**: 110203.
Zhang, H.-D., Cui, Y.-L., Huang, C., Yin, Q.-Q., Qin, X.-M., Xu, T., He, X.-F., Zhang, Y., Li, Z.-R., and Yang,

Z.-N. (2015). PPR protein PDM1/SEL1 is involved in RNA editing and splicing of plastid genes in Arabidopsis thaliana. Photosynth. Res. **126**: 311–321.
Zhang, J., Xiao, J., Li, Y., Su, B., Xu, H., Shan, X., Song, C., Xie, J., and Li, R. (2017). PDM3, a pentatricopeptide repeat-containing protein, affects chloroplast development. J. Exp. Bot. **68**: 5615–5627.
Zhang, L. et al. (2021a). Mitochondrion-targeted PENTATRICOPEPTIDE REPEAT5 is required for cis-splicing of nad4 intron 3 and endosperm development in rice. The Crop Journal **9**: 282–296.
Zhang, L., Chen, J., Zhang, L., Wei, Y., Li, Y., Xu, X., Wu, H., Yang, Z.N., Huang, J., Hu, F., Huang, W., and Cui, Y.L. (2021b). The pentatricopeptide repeat protein EMB1270 interacts with CFM2 to splice specific group II introns in Arabidopsis chloroplasts. J. Integr. Plant Biol. **63**: 1952–1966.
Zhao, P., Wang, F., Li, N., Shi, D.-Q., and Yang, W.-C. (2020). Pentatricopeptide repeat protein MID1 modulates nad2 intron 1 splicing and Arabidopsis development. Sci. Rep. **10**: 2008.
Zhao, Y., Xu, W., Zhang, Y., Sun, S., Wang, L., Zhong, S., Zhao, X., and Liu, B. (2021). PPR647 Protein Is Required for Chloroplast RNA Editing, Splicing and Chloroplast Development in Maize. Int. J. Mol. Sci. **22**.
Zheng, P. et al. (2021). OsPPR939, a nad5 splicing factor, is essential for plant growth and pollen development in rice. Theor. Appl. Genet. **134**: 923–940.
Zhu, C., Jin, G., Fang, P., Zhang, Y., Feng, X., Tang, Y., Qi, W., and Song, R. (2019). Maize pentatricopeptide repeat protein DEK41 affects cis-splicing of mitochondrial nad4 intron 3 and is required for normal seed development. J. Exp. Bot. **70**: 3795–3808.
